# Synthesis, spectroscopic, DFT calculations, biological activity, SAR, and molecular docking studies of novel bioactive pyridine derivatives

**DOI:** 10.1038/s41598-023-42714-w

**Published:** 2023-09-20

**Authors:** Kurls E. Anwer, Zeinab K. Hamza, Ramadan M. Ramadan

**Affiliations:** 1 Department of Chemistry, Faculty of Science, Ain Shams University, Cairo, Egypt; 2 Food Toxicology and Contaminants Department, National Research Centre, Giza, Egypt

**Keywords:** Chemical biology, Drug discovery, Chemistry

## Abstract

Enaminonitrile pyridine derivative was used as a precursor for preparation of fourteen heterocyclic compounds using both conventional thermal and microwave techniques. Diverse organic reagents, such as chloroacetyl chloride, acetic anhydride, chloroacetic acid, carbon disulfide, *p*-toluene sulfonyl chloride, maleic anhydride, phthalic anhydride, were used. The chemical formulae and structures of isolated derivatives were obtained using different analytical and spectroscopic techniques such as IR, ^1^H-, ^13^C-NMR as well as mass spectrometry. The spectroscopic analyses revealed diverse structure arrangements for the products. Molecular structure optimization of certain compounds were performed by the density functional theory (DFT/B3LYP) method and the basis set 6–31 G with double zeta plus polarization (d,p). The antimicrobial inhibition and the antioxidant activity of the reported compounds were screened. Compounds **5**, **6**, **11** and **13** exhibited the highest antibacterial inhibition, while compound **8** gave the highest scavenging activity (IC_50_ 43.39 µg/ml) against the DPPH radical. Structure–activity relationship of the reported compounds were correlated with the data of antibacterial and the antioxidant activity. The global reactivity descriptors were also correlated with the biological properties of compounds. The molecular docking studies of reported compounds were investigated, and the analysis showed that the docked compounds have highly negative values for the functional binding scores. The binding interaction was found to be correlated with the substituent fragments of the compounds.

## Introduction

Heterocyclic derivatives containing nitrogen such as azines and pyridine frameworks are important derivatives in both chemistry and biology, and they are commonly used in pharmaceuticals, vitamins, natural products and functional reagents syntheses^1–5^. It is known that pyridine derivatives have improved therapeutic properties. These particular properties have grafted the organic researchers to pay more attention on the key molecule with different geometries, which determine possible interactions with a specific protein or DNA, and define the biological selectivity for the target molecule^6^. Pyridine derivatives are widely reported as potential anticancer^7^, antibacterial^8^, anti-fibrotic agents^9^, anti-inflammatory^10^, cardiotonic^11^, IKK-β inhibitors^12^ and HIV-1 inhibitor^13^. From these derivatives, 2-amino-3-cyanopyridine is considered a bioactive essential supporting material to design layout structures for promising new drugs. The high reactivity of the 2-amino-3-cyanopyridine scaffold illustrated that it can be used to prepare many reactive organic intermediates used to produce nicotinamides and many pharmaceutical drugs like Ridogrel and Pirenzepine^14,15^. Furthermore, substitution of phenyl or aryl moieties in the fourth and sixth position of 2-amino-3-cyanopyridine, was found to enhance the biological activities of these products. For example, derivatives of 2-amino-3-cyano-6-(1H-indol-3-yl)-4-phenylpyridine were found to be cytotoxic against different human tumors cell. The pharmacological investigations proved that introduction indole core improved the antitumor activities of 4,6-diaryl-2-amino-3-cyanopyridines^16^. Moreover, derivatives of pyrimidine and pyridine such as substituted 2,6-diaryl- and 3,4-dihydropyrimidinone exhibited appropriate cytotoxic effects against several cancer cell lines, which reflected the potential application of these compounds to modify and develop more selective anticancer agents^17^. On the other hand, these derivatives and related ones showed very interesting spectroscopic properties and fluorescent effects, which have many important applications^18,19^.

Because of the sound biological activities of this category of derivatives, many reports appeared to develop the synthesis of novel pyridine derivatives using either conventional or microwave irradiation methods. Notably, the microwave irradiation recently gained much attention and considered as one of the preferred green chemistry tools because of its capability in improving the obtained yield and reducing the reaction times as well as it is environmentally safe^20,21^.

Our interest in synthesis of nitrogen-based heterocyclic derivatives that have prominent biological activity^20,22–24^ encouraged us to synthesize a series of pyridine derivative that could have potential bioactivity. Here, we report the synthesis of an enaminonitrile pyridine, 2-amino-6-(2,4-dimethoxyphenyl)-4-phenylnicotinonitrile, along with its reactions with some selected reagents. Different structure arrangements were concluded from the analytical and spectroscopic studies. The reported derivatives were identified by using different spectroscopic and analytical techniques along with theoretical DFT calculations and molecular docking analysis. The antimicrobial and antioxidant activities of the reported derivatives also showed that some derivatives have potent inhibition towards the screened microorganisms. Structure–activity relationships (SAR) of the different products are correlated with the biological studies and the theoretical findings.

## Experimental

### Reagents and instruments

The chemicals and reagents for this study were of analytical reagents and supplied by Sigma Aldrich. Solvents used were purified by the known standard methods. Thin layer chromatography (TLC) experiments were carried out on precoated silica gel plates purchased from Merck Kiesel gel 60F 254, BDH. Digital Stuart SMP3 electric melting point equipment was used to measure the melting points of the compounds. The microwave irradiation reactions (10 mL borosilicate glass vials) were performed using Anton Paar (monowave 300) microwave reactor. Mass spectrometry (EI, 70 eV) measurements were performed on a GC-2010 Shimadzu GCM spectrometer. Perkin-Elmer 293 spectrophotometer instrument was used for the IR measurements (KBr pellets, cm^−1^). ^1^H- and ^13^C-NMR measurements had been performed on a Varian Mercury 300 MHz spectrometer. Deuterated DMSO solutions of the samples with TMS as internal standard were used for the analysis. The microanalytical (CHN) analyses were carried out using a Perkin-Elmer analyzer (CHN-2400). Accuracy of the data were within ± 0.3% of calculated values.

### Syntheses procedures of the compounds

#### 2-Amino-6-(2,4-dimethoxyphenyl)-4-phenylnicotinonitrile (*1*)

Equimolar mixture of benzaldehyde (0.01 mol, 1.09 mL), malononitrile (0.01 mol, 0.66 g), 2,4-dimethoxyacetophenone (0.01 mol, 1.8 g) with CH_3_COONH_4_ (0.015 mol, 1.16 g) was fused at 100 °C for a time period of 5h. Reaction mixture was cooled, and the obtained residue was washed with water several times and then recrystallized using hot alcohol. Yield: Conventional heating, 70%; Microwave radiation, 93%.

#### General procedure for the synthesis of the compounds *2*–*15*.

A solution o a mixture of compound **1** (0.01 mol, 3.31 g) and the relevant reagent in the selected solvent was heated to reflux. Reaction mixture was cooled and decanted in crushed ice (~ 100 g). The precipitated residue was collected and then recrystallized from hot methanol.

** Reagents for the synthesis of compounds **2–15**.

(**2**) 2-chloro-N-(3-cyano-6-(2,4-dimethoxyphenyl)-4-phenylpyridin-2-yl)acetamide: Chloroacetyl chloride (1.12 mL)/DMF (30 mL)/18h. Yield: Conventional heating, 72%; Microwave radiation, 90%.

(**3**) 2-Cyano-N-(3-cyano-6-(2,4-dimethoxyphenyl)-4-phenylpyridin-2-yl)acetamide: Cyano acetic acid (0.85 g)/acetic anhydride (30 mL)/12h. Yield: Conventional heating, 77%; Microwave radiation, 91%.

(**4**) N-acetyl-N-(3-cyano-6-(2,4-dimethoxyphenyl)-4-phenylpyridin-2-yl)acetamide: Acetic anhydride (30 mL)/24h. Yield: Conventional heating, 71%; Microwave radiation, 93%.

(**5**) Ethyl-N-(3-cyano-6-(2,4-dimethoxyphenyl)-4-phenylpyridin-2-yl)formimidate: Triethyl orthoformate (10 mL)/20h. Yield: Conventional heating, 74%; Microwave radiation, 90%.

(**6**) 6-(2,4-Dimethoxyphenyl)-2-(methylamino)-4-phenylnicotinonitrile: Chloroacetic acid (0.94 g)/pyridine (20 mL)/19h. Yield: Conventional heating, 76%; Microwave radiation, 94%.

(**7**) 6-(2,4-Dimethoxyphenyl)-2-isothiocyanato-4-phenylnicotinonitrile: Carbon disulphide (30 mL)/28h. Yield: Conventional heating, 77%.

(**8**) N-(3-Cyano-6-(2,4-dimethoxyphenyl)-4-phenylpyridin-2-yl)-4-methylbenzenesulfonamide: *p*-Toluene sulfonyl chloride (1.9 g)/butanol (30 mL)/10h/recrystallized from hot benzene. Yield: Conventional heating, 70%; Microwave radiation, 90%.

(**9**) 6-(2,4-Dimethoxyphenyl)-2-(2,5-dioxo-2,5-dihydro-1H-pyrrol-1-yl)-4-phenylnicotinonitrile: Maleic anhydride (0.98 g)/acetic acid (30 mL)/16h. Yield: Conventional heating, 74%; Microwave radiation, 88%.

(**10**) 6-(2,4-Dimethoxyphenyl)-2-(1,3-dioxoisoindolin-2-yl)-4-phenylnicotinonitrile: Phthalic anhydride (1.48 g)/acetic acid (30 mL)/17h. Yield: Conventional heating, 72%; Microwave radiation, 92%.

(**11**) 7-(2,4-Dimethoxyphenyl)-5-phenylpyrido[2,3-d]pyrimidin-4-amine: Formamide (30 mL)/20h. Yield: Conventional heating, 73%; Microwave radiation, 87%.

(**12**) 4-amino-7-(2,4-dimethoxyphenyl)-2,5-diphenyl-1,8-naphthyridine-3-carbonitrile: Benzylidene malononitrile (1.54 g)/butanol (30 mL)/24h/recrystallized from hot dioxane. Yield: Conventional heating, 77%; Microwave radiation, 89%.

(**13**) 4-Amino-7-(2,4-dimethoxyphenyl)-2-hydroxy-5-phenyl-1,8-naphthyridine-3-carbonitrile: Ethyl cyanoacetate (1.13 mL)/drops of piperidine/DMF (30 mL)/17h. Yield: Conventional heating, 73%; Microwave radiation, 87%.

(**14**) 2,4-Diamino-7-(2,4-dimethoxyphenyl)-5-phenyl-1,8-naphthyridine-3-carbonitrile: Malononitrile (0.66 g)/drops of piperidine/butanol (30 mL)/10h. Yield: Conventional heating, 71%; Microwave radiation, 90%.

(**15**) 7-(2,4-Dimethoxyphenyl)-2-methyl-5-phenylpyrido[2,3-d]pyrimidin-4(3H)-one: Few drops of conc. sulphuric acid/acetic anhydride (30 mL)/15h/recrystallized from hot acetone. Yield: Conventional heating, 72%; Microwave radiation, 90%.

Table 1 gives the melting points, elemental analyses and mass spectral data for all derivatives.

**Table 1:** Elemental analyses and mass spectrometry data for the reported compounds.

Compound	m.p., ^o^C	Elemental analysis, Found (Calcd.)			Mass spectrometry	
		% C	% H	% N	M.M	m/z
C_20_H_17_N_3_O_2_ **(1)**	230–232	72.27 (72.49)	5.06 (5.17)	12.72 (12.68)	331.38	331 [P^+^] (24.92%)
C_22_H_18_ClN_3_O_3_ (2)*	150–152	64.83 (64.79)	4.38 (4.45)	10.41 (10.30)	407.85	407 [P^+^] (13.05%)
C_23_H_18_N_4_O_3_ **(3)**	188–190	69.25 (69.34)	4.61 (4.55)	14.23 (14.06)	398.42	398 [P^+^] (20.26%)
C_24_H_21_N_3_O_4_ **(4)**	146–148	69.21 (69.39)	5.47 (5.10)	10.16 (10.11)	415.45	415 [P^+^] (20%)
C_23_H_21_N_3_O_3_ **(5)**	> 300	71.15 (71.30)	5.29 (5.46)	10.92 (10.85)	387.44	387 [P^+^] (24%)
C_21_H_19_N_3_O_2_ **(6)**	250–252	73.12 (73.03)	5.41 (5.54)	12.02 (12.17)	345.40	345 [P^+^] (18.05%)
C_21_H_15_N_3_O_2_S (7)**	> 300	67.35 (67.54)	4.14 (4.05)	11.07 (11.25)	373.43	373 [P^+^] (55.07%)
C_27_H_23_N_3_O_4_S (8)***	> 300	66.68 (66.79)	4.82 (4.77)	8.77 (8.65)	485.56	485 [P^+^] (18.0%)
C_24_H_17_N_3_O_4_ **(9)**	168–170	70.16 (70.07)	4.28 (4.17)	10.39 (10.21)	411.42	411 [P^+^] (46.77%)
C_28_H_19_N_3_O_4_ **(10)**	280–282	72.98 (72.88)	4.03 (4.15)	9.07 (9.11)	461.48	461 [P^+^] (9.29%)
C_21_H_18_N_4_O_2_ **(11)**	> 300	70.42 (70.38)	5.14 (5.06)	15.42 (15.63)	358.40	358 [P^+^] (23.33%)
C_29_H_22_N_4_O_2_ **(12)**	> 300	75.88 (75.97)	4.58 (4.84)	12.31 (12.22)	458.52	458 [P^+^] (15.8%)
C_23_H_18_N_4_O_3_ **(13)**	> 300	69.42 (69.34)	4.46 (4.55)	14.12 (14.06)	398.42	398 [P^+^] (12.43%)
C_23_H_19_N_5_O_2_ **(14)**	192–194	69.59 (69.51)	4.75 (4.82)	17.66 (17.62)	397.44	397 [P^+^] (16.25%)
C_22_H_19_N_3_O_3_ (**15)**	262–264	70.68 (70.76)	5.02 (5.13)	11.44 (11.25)	373.41	373 [P^+^] (14.47%)

*% Cl, 8.51 (8.69); **% S, 8.64 (8.59); ***% S, 6.59 (6.60).

### Molecular orbital calculations

Molecular orbital computation was performed using the software package of Gaussian 09W^25^. DFT (B3LYP) method and the basis set 6-31G (d,p) with double zeta plus polarization for carbon, hydrogen, nitrogen, oxygen, sulfur and chloride was applied.

### Biological activity studies

#### Antimicrobial activity

In vitro evaluation of the reported compounds for their antimicrobial activities (agar well diffusion method) was performed. The experimental procedures of the studies were as described in previous report^26^. The antibacterial screening of the compounds was against three gram-negative bacteria (*Escherichia coli*, *Salmonella typhimurium* and *Yersinia enterocolitica*) and two gram-positive bacteria (*Staphylococcus aureus* and *Bacillus cereus*). For the antibacterial activity, Cefoperazone was served as a standard. The antifungal activities were checked on three fungi (*Aspergillus flavus*, *Aspergillus niger* and *Fusarium oxysporum*). Analyses for every compound were performed in triplicate measurements and the average values are reported.

#### Antioxidant study

Scavenging modeling of the stable radical 2,2-diphenyl-1-picrylhydrazyl (DPPH) is employed to determine antioxidant activity of tested derivatives^26^. 1.0 mM stock solution of DPPH was prepared in methanol. Solutions of different concentrations (25–200 µg mL^−1^) from ascorbic acid and the tested compounds were prepared using DMSO. 1.0 mL of the sample solution was mixed with 3.0 mL (0.1 mM) of DPPH. Incubation of the samples at room temperature for 30 min. Control experiment was performed without the tested samples. Measurements of the absorbance at 517 nm of the tested solutions were performed. As the concentration of the tested compounds increased, a marked decrease in the absorption was observed. This markable decrease indicated strong antioxidant activity of the compounds. The DPPH was a positive control, DMSO was a negative control and ascorbic acid was a standard. Reduction of DPPH was estimated relative to measured absorbance of the control. The measurements were carried out in triplicates. Percentage of the radical scavenging was computed using the relation:

$$ \% {\text{Radical}}\;{\text{scavenging}}\;{\text{activity}} = \frac{{{\text{A}}_{{\text{c}}} - {\text{A}}_{{\text{s}}} }}{{{\text{A}}_{{\text{s}}} }} $$

Absorbance of control reaction having all reagents excepting the tested compounds is given A_C_. Absorbance of sample at any concentration C is denoted A_S_. Effective concentrations of the sample desired for scavenging 50% (IC_50_ value) of DPPH radical was obtained by plotting the percentage of inhibition versus concentrations with the use of linear regression analysis.

### Molecular docking studies

Docking studies were carried out using Molecular Operating Environment (MOE) package version 2014.0901. Structure of a B-DNA (X-ray crystal structure) with the code PDB ID: 1BNA was downloaded from Research Collaboratory for Structural Bioinformatics (RCSB) database.

## Results and discussion

### The synthesis of compounds and spectroscopic studies

**Figure 1:**
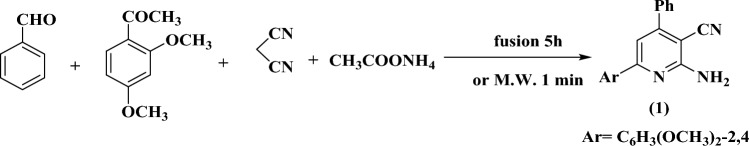
Synthesis of the precursor compound** 1**.

**Figure 2:**
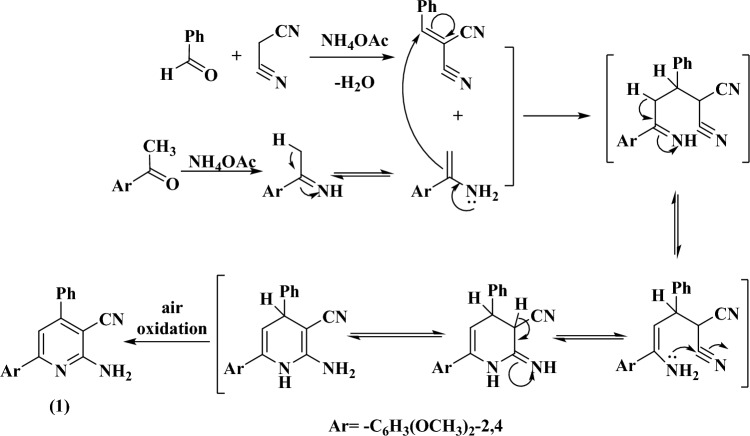
Expected mechanism for formation of compound** 1**.

The enaminonitrile pyridine derivative, 2-amino-6-(2,4-dimethoxyphenyl)-4-phenylnicotinonitrile (**1**), was prepared using either thermal or microwave irradiation techniques. It was proceeded via a four-component one-pot reaction including benzaldehyde, 2,4-dimethoxy acetophenone, malononitrile, and ammonium acetate (Fig. 1). Structure of **1** was elucidated by elemental analyses and mass spectrometry (Table 1) and the IR, ^1^H- and ^13^C-NMR spectroscopic tools (Table 2). Compound **1** displayed a signal at m/z 331 in its mass spectrum due to the molecular peak. Infrared spectrum of **1** displayed the different functional groups in their expected positions. It exhibited two asymmetric and symmetric stretching frequencies for NH_2_ group at 3460 and 3369 cm^−1^. In addition, a strong vibrational stretching frequency at 2207 cm^−1^ was assigned for the C≡N moiety^27^. In the ^1^H-NMR spectrum of **1**, the NH_2_ protons exhibited a broad signal at 7.26 ppm, Fig. 1S**(A)**. This signal disappeared on addition of D_2_O to the solution and indicated that the protons of NH_2_ are exchangeable with the deuterium of D_2_O, Fig. 1S**(B)**. Furthermore, the^1^H-NMR spectrum of **1** showed signals due to the OCH_3_ and aromatic protons. The ^13^C-NMR spectrum of **1**, on the other hand, showed a set of signals due to carbon atoms in their appropriate positions (Fig. 2S and Table 2). As expected, the signals of the two OCH_3_ carbon atoms appeared at higher field (56.4 and 58.6 ppm). In addition, the signals of the pyridine carbon attached to the C≡N group and the signal of the cyano carbon itself also occurred at their anticipated positions (109.8 and 112.4 ppm). Figure 2 displayed a speculated mechanism for formation of the precursor **1**.

**Table 2:** IR and NMR data for the reported compounds.

Compound	IR data (cm^−1^)	NMR data, DMSO-*d*_*6*_, δ (ppm)	
		^1^H-NMR	^13^C-NMR
**1**	3460 (ʋas_NH2_), 3369 (ʋs_NH2_), 2207 (ʋ_CN_), 1602 (ʋ_C=N_)	3.81 (s, 3H, OCH_3_), 3.88 (s, 3H, OCH_3_), 6.63–8.03 (m, 9H, Ar–H), 7.26 (s, 2H, NH_2_, exchangeable with D_2_O)	56.4, 58.6, 109.8, 112.4, 115.4, 126.6, 126.8, 126.9, 127.2, 128.3, 128.8, 129.0, 131.3, 135.6, 138.9, 144.5, 152.9, 155.1, 157.2, 157.9
**2**	3198 (ʋ_NH_), 2225 (ʋ_CN_), 1727 (ʋ_CO_), 1609 (ʋ_C=N_)	3.79 (s, 3H, OCH_3_), 3.82 (s, 3H, OCH_3_), 4.19 (s, 2H, COCH_2_Cl), 6.48–8.01 (m, 9H, Ar–H), 10.47 (s, 1H, NH, exchangeable with D_2_O)	46.4, 56.3, 58.7, 109.6, 112.7, 116.0, 126.6, 126.8, 126.9, 127.2, 128.3, 128.8, 129.0, 131.2, 135.6, 138.8, 144.4, 152.9, 155.1, 157.2, 157.9, 166.9
**3**	3174 (ʋ_NH_), 2221, 2207 (ʋ_CN_), 1726 (ʋ_CO_), 1606 (ʋ_C=N_)	3.84 (s, 2H, COCH_2_CN), 3.86 (s, 3H, OCH_3_), 3.89 (s, 3H, OCH_3_), 6.69–7.89 (m, 9H, Ar–H), 8.20 (s, 1H, NH, D_2_O exchangeable)	26.8, 56.3, 58.6, 109.7, 112.7, 116.0, 123.7, 126.6, 126.8, 126.9, 127.2, 128.3, 128.8, 129.0, 131.2, 135.6, 138.8, 144.4, 152.9, 155.1, 157.2, 157.9, 166.9
**4**	IR (cm^−1^): 2221 (ʋ_CN_), 1728, 1704 (ʋ_CO_), 1606 (ʋ_C=N_)	2.17 (s, 3H, COCH_3_), 2.23 (s, 3H, COCH_3_), 3.82 (s, 3H, OCH_3_), 3.85 (s, 3H, OCH_3_), 7.13–7.96 (m, 9H, Ar–H)	26.5, 58.6, 60.1, 115.4, 121.2, 121.3, 121.5, 126.3, 126.9, 129.0, 126.1, 129.7, 129.8, 131.3, 131.9, 132.4, 1344, 137.7, 138.0, 152.6, 153.1, 156.8, 157.1, 172.0
**5**	IR (cm^−1^): 2221 (ʋ_CN_), 1606 (ʋ_C=N_)	1.41 (d, 3H, J = 8 Hz, OCH_2_CH_3_), 3.84 (s, 3H, OCH_3_), 3.87 (s, 3H, OCH_3_), 4.55 (t, 2H, J = 8.6 Hz, OCH_2_CH_3_), 6.69–99 (m, 9H, Ar–H), 8.44 (s, 1H, N=CH)	14.2, 60.2, 60.3, 108.1, 111.7, 120.3, 125.8, 126.1, 128.3, 1286.6, 128.7, 130.0, 131.3, 133.1, 133.7, 135.9, 142.6, 145.4, 152.7, 155.4, 164.2
**6**	IR (cm^−1^): 3104 (ʋ_NH_), 2204 (ʋ_CN_), 1596 (ʋ_C=N_)	2.78 (s, 3H, NHCH_3_), 3.79 (s, 3H, OCH_3_), 3.84 (s, 3H, OCH_3_), 7.06–8.11 (m, 9H, Ar–H), 9.75 (s, 1H, NH)	30.2, 56.4, 58.6, 109.8, 112.4, 115.4, 126.6, 126.8, 126.9, 127.2, 128.3, 128.8, 129.0, 131.3, 135.6, 138.9, 144.5, 152.9, 155.1, 157.2, 157.9
**7**	IR (cm^−1^): 2220 (ʋ_CN_), 1596, 1571 (ʋ_C=N_), 1492 (ʋ_C=S_)	3.82 (s, 3H, OCH_3_), 3.86 (s, 3H, OCH_3_), 6.70–8.05 (m, 9H, Ar–H)	56.4, 58.6, 108.7, 111.2, 115.3, 126.6, 126.8, 126.9, 127.1, 128.3, 128.6, 129.0, 132.3, 135.6, 138.9, 140.4, 144.5, 151.9, 155.2, 156.8, 157.9
**8**	IR (cm^−1^): 3204 (ʋ_NH_), 2223 (ʋ_CN_), 1649 (ʋ_S=O_), 1605 (ʋ_C=N_)	2.71 (s, 3H, CH_3_), 3.83 (s, 3H, OCH_3_), 3.87 (s, 3H, OCH_3_), 6.71–7.87 (m, 13H, Ar–H), 10.76 (s, 1H, NH)	21.3, 55.8, 56.1, 85.6, 98.8, 107.1, 111.1, 111.7, 113.7, 114.4, 127.4, 128.3, 129.2, 129.3, 132.6, 137.6, 136.7, 139.0, 154.4, 158.3, 158.4, 160.2, 165.3
**9**	IR (cm^−1^): 2224 (ʋ_CN_), 1763, 1723 (ʋ_C=O_), 1608 (ʋ_C=N_)	3.78 (s, 3H, OCH_3_), 3.87 (s, 3H, OCH_3_), 6.64–8.21 (m, 11H, Ar–H)	60.2, 60.3, 111.7, 125.8, 126.1, 128.3, 128.5, 128.7, 130.4, 131.3, 133.1, 133.7, 135.9, 142.7, 145.4, 152.5, 153.0, 157.2, 157.9, 164.5, and 164.9
**10**	IR (cm^−1^): 2224 (ʋ_CN_), 1789, 1730 (ʋ_C=O_), 1609 (ʋ_C=N_)	3.81 (s, 3H, OCH_3_), 3.88 (s, 3H, OCH_3_), 6.65–8.23 (m, 13H, Ar–H)	58.3, 58.7, 109.8, 110.8, 111.0, 113.6, 113.8, 116.0, 117.0, 117.9, 119.5, 120.2, 120.4, 123.8, 129.3, 130.4, 130.8, 144.0, 148.5, 148.6, 151.4, 162.5, 162.9
**11**	IR (cm^−1^): 3369 (ʋas_NH2_), 3219 (ʋs_NH2_), 1610 (ʋ_C=N_)	3.76 (s, 3H, OCH_3_), 3.83 (s, 3H, OCH_3_), 6.56–8.52 (m, 10H, Ar–H), 11.37 (s, 2H, NH_2_)	55.8, 56.1, 98.8, 106.7, 107.1, 114.4, 119.3, 127.4, 129.2, 132.6, 143.5, 150.2, 151.2, 155.0, 157.3, 157.4, 158.4, 160.2
**12**	IR (cm^−1^): 3369 (ʋas_NH2_), 3219 (ʋs_NH2_), 2201 (ʋ_CN_), 1610 (ʋ_C=N_)	3.83 (s, 3H, OCH_3_), 3.87 (s, 3H, OCH_3_), 6.70–8.04 (m, 14H, Ar–H), 10.32 (s, 2H, NH_2_, D_2_O Exchangeable)	60.2, 60.3, 125.8, 126.1, 128.3, 128.6, 128.7, 130.4, 131.3, 133.1, 133.7, 135.9, 142.7, 145.4, 164.2, 164.5, 164.9, 168.0, 169.4
**13**	IR (cm^−1^): 3423 (ʋ_OH&NH2_, broad), 2204 (ʋ_CN_), 1596 (ʋ_C=N_)	3.52 (s, 2H, NH_2_, D_2_O Exchangeable), 3.86 (s, 3H, OCH_3_), 3.96 (s, 3H, OCH_3_), 6.70–8.04 (m, 9H, Ar–H), 12.58 (s, 1H, OH, D_2_O Exchangeable)	55.8, 56.1, 85.0, 93.5, 98.8, 107.1, 114.4, 114.8, 119.3, 127.4, 129.2, 132.6, 143.5, 150.2, 152.9, 155.0, 158.4, 158.8, 160.2, 165.8
**14**	IR (cm^−1^): 3460, 3363, 3221 (ʋ_NH2_), 2203 (ʋ_CN_), 1616 (ʋ_C=N_)	3.82 (s, 3H, OCH_3_), 3.86 (s, 3H, OCH_3_), 6.65–8.01 (m, 9H, Ar–H), 7.20 (s, 2H, NH_2_, D_2_O exchangeable), 9.28 (s, 2H, NH_2_, D_2_O exchangeable)	55.8, 56.1, 77.4, 98.8, 107.1, 114.4, 114.5, 118.4, 119.3, 127.4, 129.2, 132.6, 143.5, 150.2, 155.0, 156.6, 158.4, 160.2, 162.0, 162.2
**15**	IR (cm^−1^): 2991 (ʋ_NH_), 1710 (ʋ_CO_), 1610 (ʋ_C=N_)	2.36 (s, 3H, N=CCH_3_), 3.82 (s, 3H, OCH_3_), 3.86 (s, 3H, OCH_3_), 6.70–8.05 (m, 9H, Ar–H), 11.51 (s, 1H, NH, exchangeable with D_2_O)	21.8, 56.5, 58.6, 112.4, 115.5, 126.6, 126.9, 126.8, 127.3, 128.3, 128.7, 129.0, 131.2, 135.7, 138.9, 144.4, 152.5, 155.2, 157.1, 157.8, 168.9

**Figure 3:**
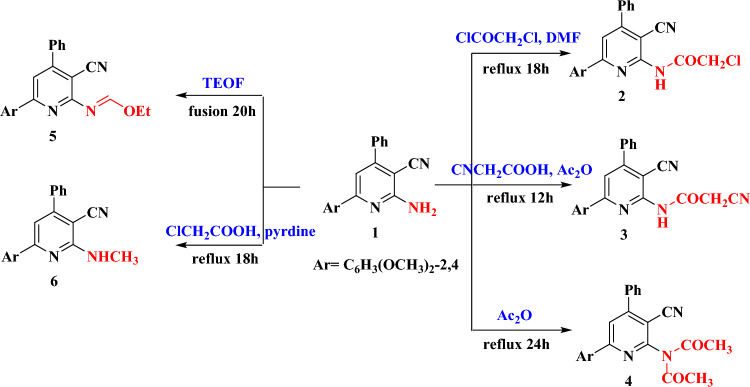
Synthesis and structure of compounds **2**–**6**.

It was found that compound **1** is a reactive derivative and can react with a diverse number of reagents. It reacted with either chloroacetyl chloride, cyanoacetic acid or acetic anhydride to give 2-chloro-acetamide, 2-cyano-acetamide, and N-acetyl-acetamide pyridine derivatives (**2**–**4**), respectively (Fig. 3). Interestingly, formation of compound **4** in a refluxing triethyl orthoformate (TEOF) in presence of acetic anhydride was the predominant rout. Structures of the isolated derivatives were supported by using the elemental analysis, IR, ^1^H- and ^13^C-NMR as well as their mass spectra (Tables 1 and 2). Although the two derivatives **2** and **3** are quite similar in structure, it is worth mentioning that they displayed NH signals in their ^1^H NMR spectra at different positions (10.47 and 8.20 ppm, respectively). The higher downfield shift of the NH signal of **2** could be presumably due to the Cl atom in the attached moiety COCH_2_Cl. In addition, all the three compounds **2–4** showed signals around 3.8 ppm due to the OCH_3_ attached to the arene ring. Notably, the derivative **4** displayed in addition two more higher field signals at 2.17 and 2.23 ppm due to the OCH_3_ groups that substituted to the NH_2_ group (Fig. 3). The ^13^C NMR of compound **3** illustrated interesting features. The spectrum showed two distinct C≡N groups. The one attached directly to pyridine ring with value like the parent compound (112.7 ppm). The other group, which bonded to the HNCOCH_2_ moiety exerted downfield shift and appeared at 126.6 ppm. The carbonyl carbon in that group occurred at 166.9 ppm like that of compound **2**.

**Figure 4:**
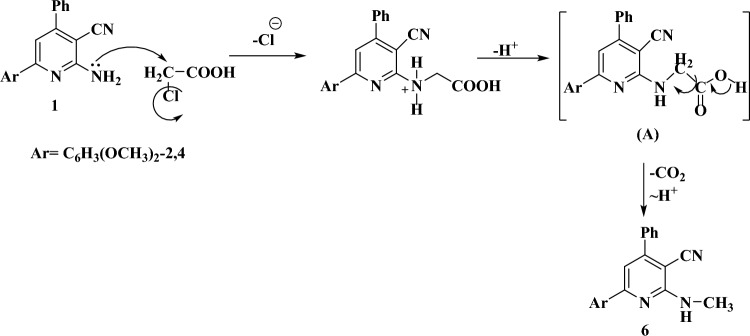
Proposed mechanism for the formation of compound **6**.

The formimidate derivative **5** was obtained by solid fusion of compound **1** with TEOF for a period of 20h (Fig. 3). Structure of **5** was illustrated using spectroscopic and analytical measurements. Compound **5** demonstrated a peak at m/z = 387 in its mass spectrum due to the molecular formula. Thus, the spectroscopic finding were compatible with the suggested structure. On the other hand, as shown from Fig. 3, compound **1** reacted with chloroacetic acid in pyridine to give an N-methyl amine derivative (**6**). As declared from Fig. 4, derivative **6** might be occurred via nucleophilic attack of NH_2_ group of **1** on the CH_2_ group of chloroacetic acid with elimination of one mole of HCl to form the intermediate (A), then followed by decarboxylation^28,29^. Decarboxylation of the COOH in position 2 of pyridine ring could easily occur due to the high probability to form intramolecular hydrogen bonding between H of the carboxylic group and the lone pair of N atom of pyridine. Notably, the product did not give effervescence with the acidity test confirming the absence of the carboxylic group. Compound **6** displayed a signal at m/z at 345 in its mass spectrum due to the molecular formula. The IR spectrum was devoid from bands due to the C=O and OH groups. Instead, it displayed one band due to the NH group (3104 cm^−1^). On the other hand, the ^1^H-NMR spectrum of the **6** exhibited a singlet with integration of three hydrogens (δ = 2.78 ppm) due to the methyl group of the NHCH_3_ part. In addition, the spectrum exhibited a signal at 9.75 ppm for the NH proton.

**Figure 5:**
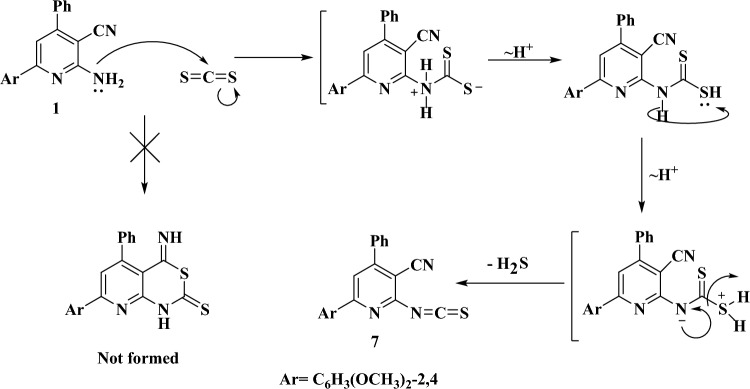
Proposed pathway for formation of compound **7**.

The precursor compound **1** reacted with carbon disulfide to afford the unexpected isothiocyanate pyridine derivative **7**, and not the familiar pyridothiazinethione as we previously reported^30^. Figure 5 illustrates a proposed pathway for the formation of compound **7**. The last step in the reaction involved the elimination of H_2_S gas, which was recognized by its known smell. The mass spectrum of the compound (m/z = 373, parent ion peak) as well as its elemental analysis (Table 1) confirmed the proposed chemical formula. Structure of compound **7** was indicated from the disappearing of the stretching frequency bands of the NH_2_ group in its IR spectrum as well as the occurrence of a new strong band specific for thiocarbonyl (C=S) group at 1492 cm^−1^, along with bands due to the still present C≡N and the new C=N groups (Table 2). The structure of the compound was also confirmed from its ^1^H- and ^13^C NMR spectra. The proton NMR spectrum displayed no signals due to the NH group, which would be expected if the pyridothiazinethione was the product. Moreover, the ^13^C NMR spectrum of **7** showed a signal for the C≡N group in the normal range (111.2 ppm) similar to that of the parent compound. In addition, the spectrum displayed a signal at 135.6 ppm due to the carbon atom of the N=C=S moiety^31^.

**Figure 6:**
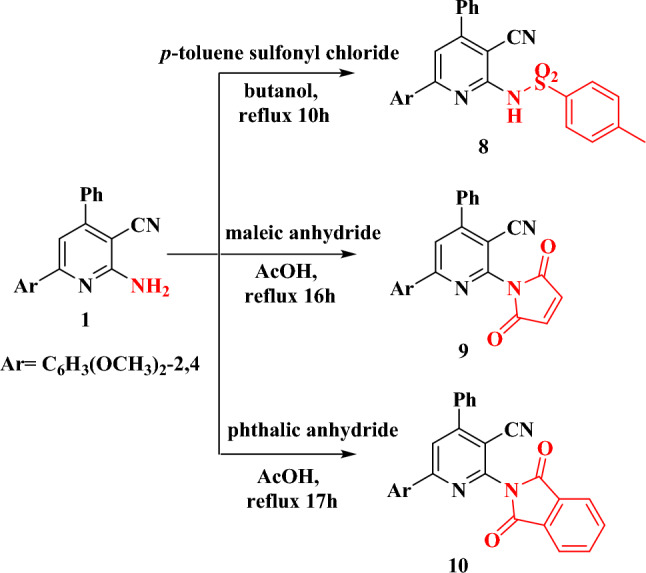
The synthesis and structure of compounds **8**–**10**.

Compound **1** interacted with *p*-toluene sulfonyl chloride, maleic anhydride, and phthalic anhydride to produce N- sulfonamide, 2,5-dioxo pyrrole, and 1,3-dioxoisoindoline pyridine derivatives (**8–10**), respectively (Fig. 6). The structures and molecular formulae of the compounds **8–10** were indicated from their spectroscopic measurements and elemental analysis. The IR spectra of the compounds showed the existence of the C≡N group at its normal range (Table 2). In addition, derivative **8** displayed an asymmetric stretching frequency band at 1649 cm^−1^ with a shoulder due to the symmetric stretching. It also showed a band due to the NH group at 3204 cm^−1^. The presence of the NH group in **8** was also confirmed from its ^1^H NMR spectrum (10.76 ppm). On the other hand, the IR spectra of the two compounds **9** and **10** displayed strong band at 1723 cm^−1^ characteristic for the carbonyl groups. The ^13^C NMR spectra of the three derivatives (**8–10**) illustrated the different carbon signal at their appropriate positions.

**Figure 7:**
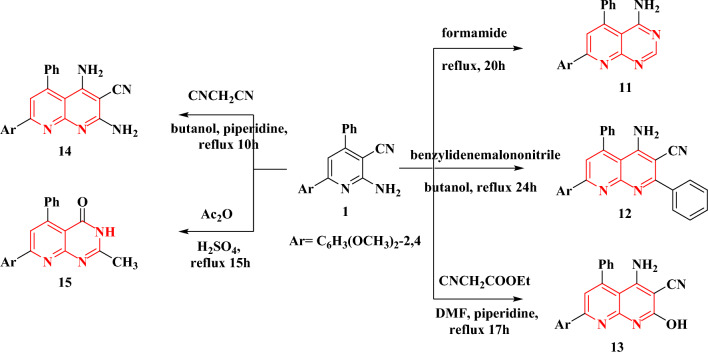
The syntheses and structures of compounds **11**–**15**.

**Figure 8:**
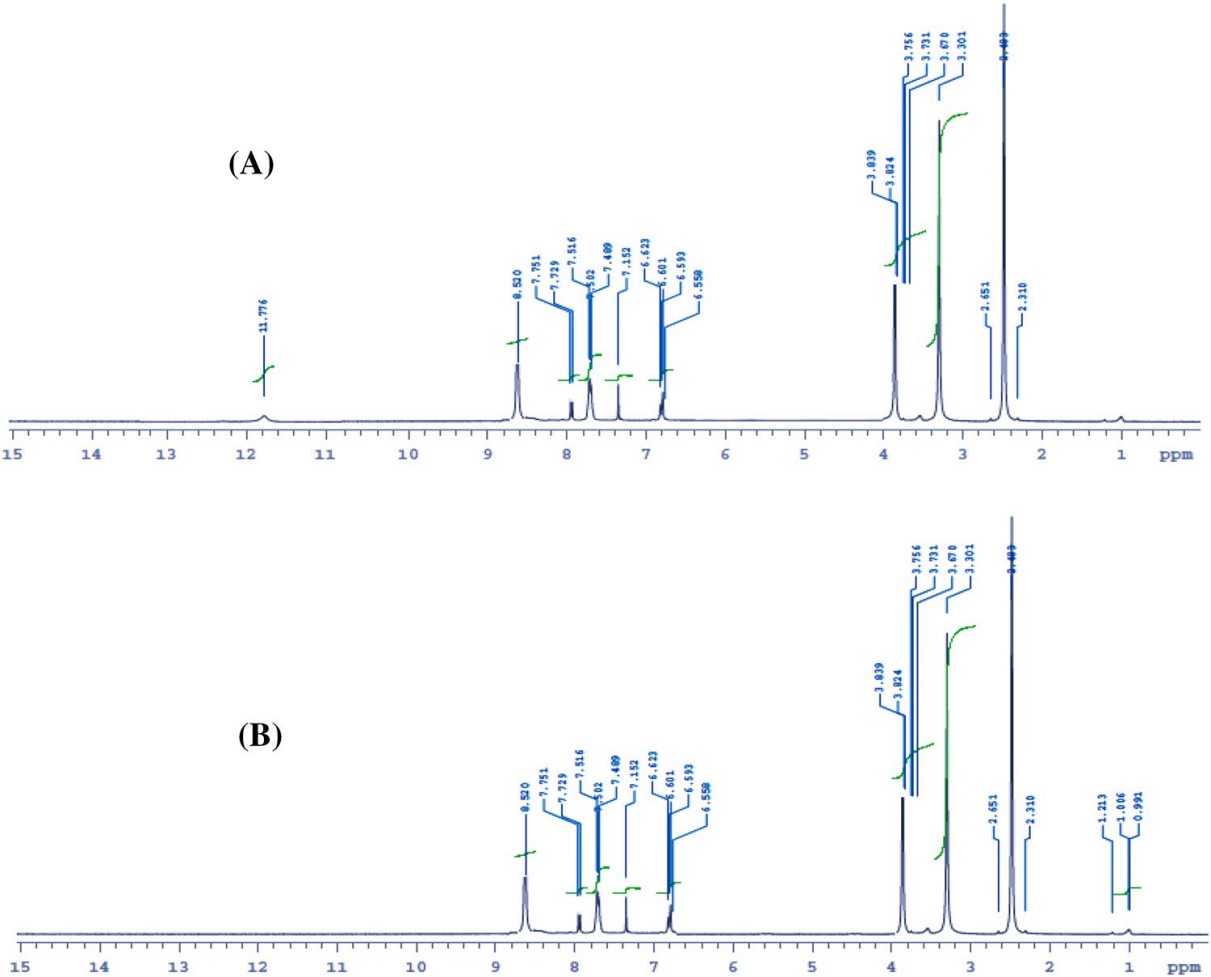
(**A**) The ^1^H-NMR spectrum of **11**; (**B**) The ^1^H-NMR spectrum of **11** + D_2_O.

The interesting pharmacological properties of naphthyridine derivatives as an antioxidant^32^, anti-inflammatory^33^, and antimicrobial^34^ reagents have prompted us to attempt to synthesize novel naphthyridine derivatives through the reaction of the bifunctional compound **1** with either formamide, benzylidene malononitrile, ethyl cyanoacetate or malononitrile using a catalytic amount of piperidine; reaction of Ac_2_O with compound **1** occurred in presence of few drops of H_2_SO_4_, (compounds **11–15**, Fig. 7). The molecular formulae and chemical structure of these new naphthyridine derivatives were elucidated using elemental analyses and spectroscopic data (Table 2). The IR spectrum of the three derivatives **11–14** indicated the presence of the NH_2_ functional group as they showed asymmetric and symmetric stretching frequencies due to the group (Table 2). The presence of the NH_2_ group was further confirmed from the ^1^H NMR spectra of the derivatives; NH_2_ the signal was disappeared on adding D_2_O to the NMR solution (Fig. 8). On the other hand, the existence of OH group in compound **13** and the NH group in compound **15** were also pointed out from their IR and ^1^H NMR spectra (Table 2). Moreover, the ^13^C NMR spectra of the four derivatives are consistent with the proposed structures.

### Stereochemistry and reactivity descriptors calculations

**Figure 9:**
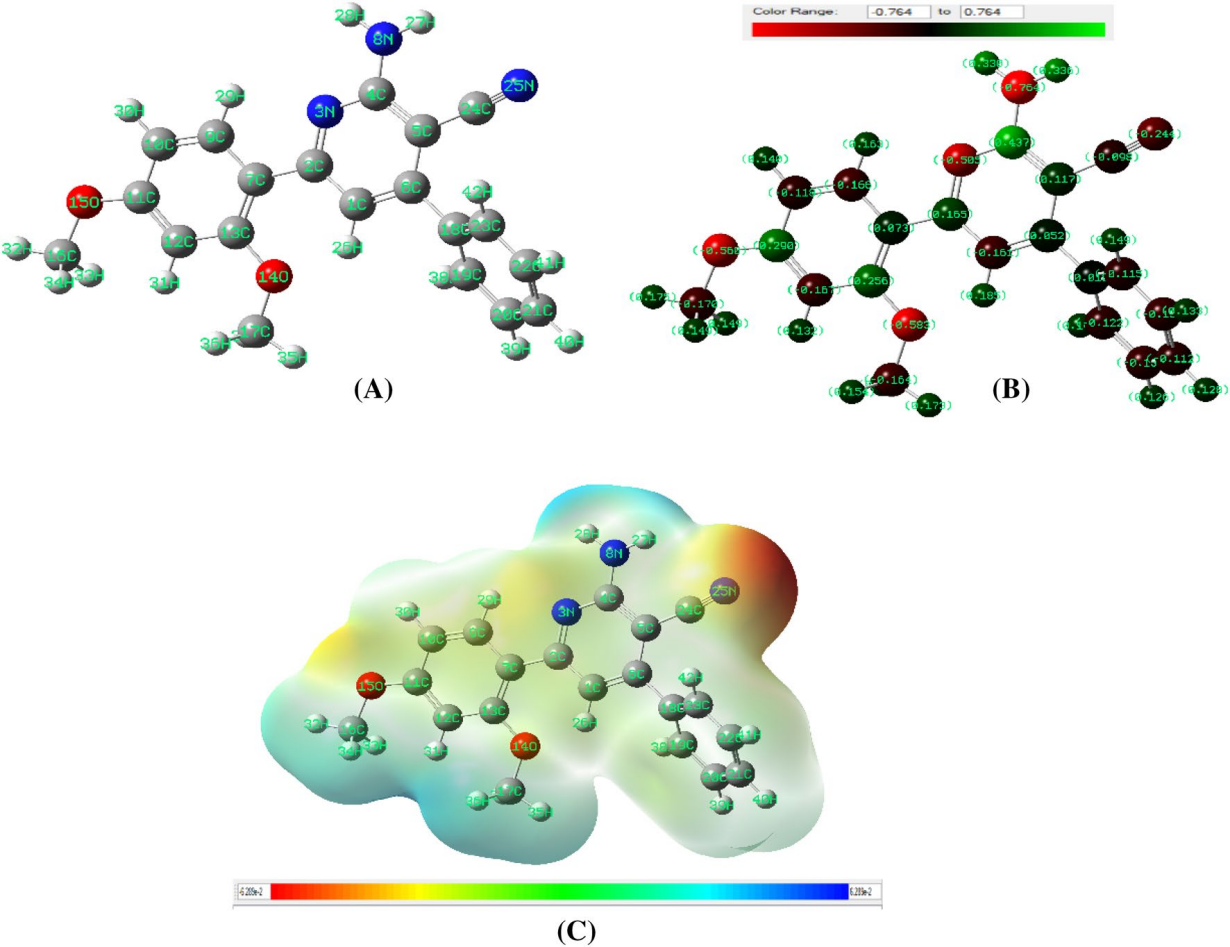
(**A**) The optimize geometrical structure of compound **1**; (**B**) The charge distribution on atoms; (**C**) Molecular electrostatic potential map.

The energetic optimize geometrical structure parameters, and steric energies of the compound **1** and its derivatives** 2**–**8**,** 11**,** 13** and **15** were computed using hybrid DFT method^25^. The *HOMO* and *LUMO* orbitals in the molecular orbital diagram of a compound explore important information in examining its optical and electric properties as well as the interaction with other entities. Further, the frontier orbitals are responsible for having the different charge transfer (CT) models^35–37^. The lowest energy minimized stable orientations of the compounds were examined by concentrating on the configuration of the function groups and their orientation relative to each other. The energetically minimized structure of compound **1** (43.31 kcal/mol) displayed specified structure features. The molecule was non-planer and unsymmetrical and having a point group of *C*_1_ (Fig. 9). For example, the pyridine ring, amino and cyano groups are lying in one plane, which is forming a dihedral angle = 43.0° with the phenyl ring attached at the fourth site of pyridine cycle. Interestingly, the two hydrogens of the –NH_2_ group were directed towards the nitrogen atoms of pyridine ring and cyano group forming some sort of hydrogen bonding with van der Waal displacement equals 2.46 Å (N3–H28) and 2.82 Å (H27–N25), respectively (Fig. 9). The of bond lengths and angles were in normal values^38–40^. The charge allocation on the different atoms of **1** calculated by Mullikan method is illustrated in Fig. 9B. According to the charge distribution shown, the nitrogen of the amino group (N8) displayed highest negative charge density (− 0.76), while nitrogen atoms of pyridine (N3) and cyano moiety (N25) have lower values (− 0.51 and − 0.24, respectively). Furthermore, the electrostatic potential (ESP) map of derivative **1** (Fig. 9C) gives important information about the charge distributions of the compound. It distinguishes the reactive sites of the tested compound by showing both the electrophilic and/or the nucleophilic attack regions. In addition, the electrostatic potential map is an effective representation in biological sciences fields^38^. It determines the positive (shown as a blue color) and negative (appeared a a red color) charged electrostatic potential in the derivative. The color scale of **1** ranged from − 0.06289 Hartree (− 165.12 kJ/mol) to + 0.06289 Hartree (+ 165.12 kJ/mol). High negative potential red zones indicated the electrophilic attack sites, while high positive potential blue sections designated appropriate centers for nucleophilic attack. It is well known that derivatives of 2-amino-3-cyanopyridine are polyfunctional compounds that possess properties for susceptible electrophilic and nucleophilic reactions. The perfect nucleophilic location in these derivatives is the NH_2_, while the carbon atom of the cyano group is liable to the electrophilic attack. Thus, several heterocyclic moieties with variable ring sizes like pyrrole, pyridine, pyrimidine and oxdiazine were designed and synthesized using these chemical properties^13^.

**Figure 10:**
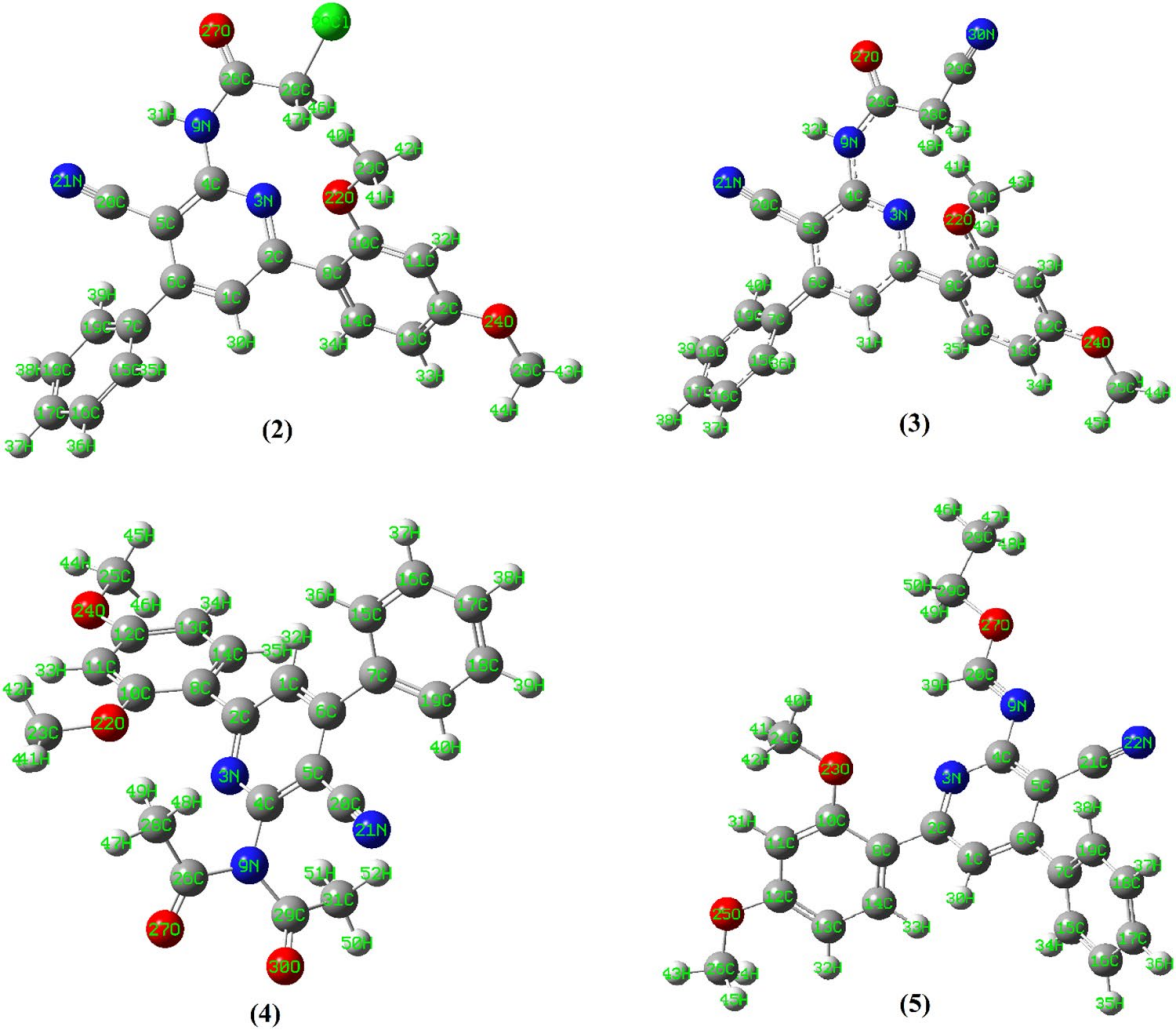
Geometrically optimize structures of compounds **2**–**5.**

The geometrically optimize structural arrangements of compounds **2**–**5** (Fig. 10) possessed steric minimize energies = 46.93, 46.09, 85.19 and 56.10 kcal/mol, respectively. The structures are unsymmetrical and non-planar (*C*_1_ point group). The non-planarity characteristics are stemmed from the dihedral angle values occurred between the plane of pyridine cycle and the two phenyl parts. All the dihedral angles are found in the range 44° and 45°, respectively. Also, all the bond distances and bond angles have similar values as shown in compound **1**. On the other hand, the pyridine nitrogen performed intramolecular hydrogen bonding with the adjacent hydrogens of the substituents in position two (**2**: N3–H47 = 2.35 Å; **3**: N3–H48 = 2.30 Å; **4**: N3–H49 = 2.56 Å; **5**: N3–H39 = 2.44 Å), Fig. 10. The presence of these hydrogen bonding was also indicated from the broadening of protons signals in ^1^H-NMR spectra of molecules.

**Figure 11:**
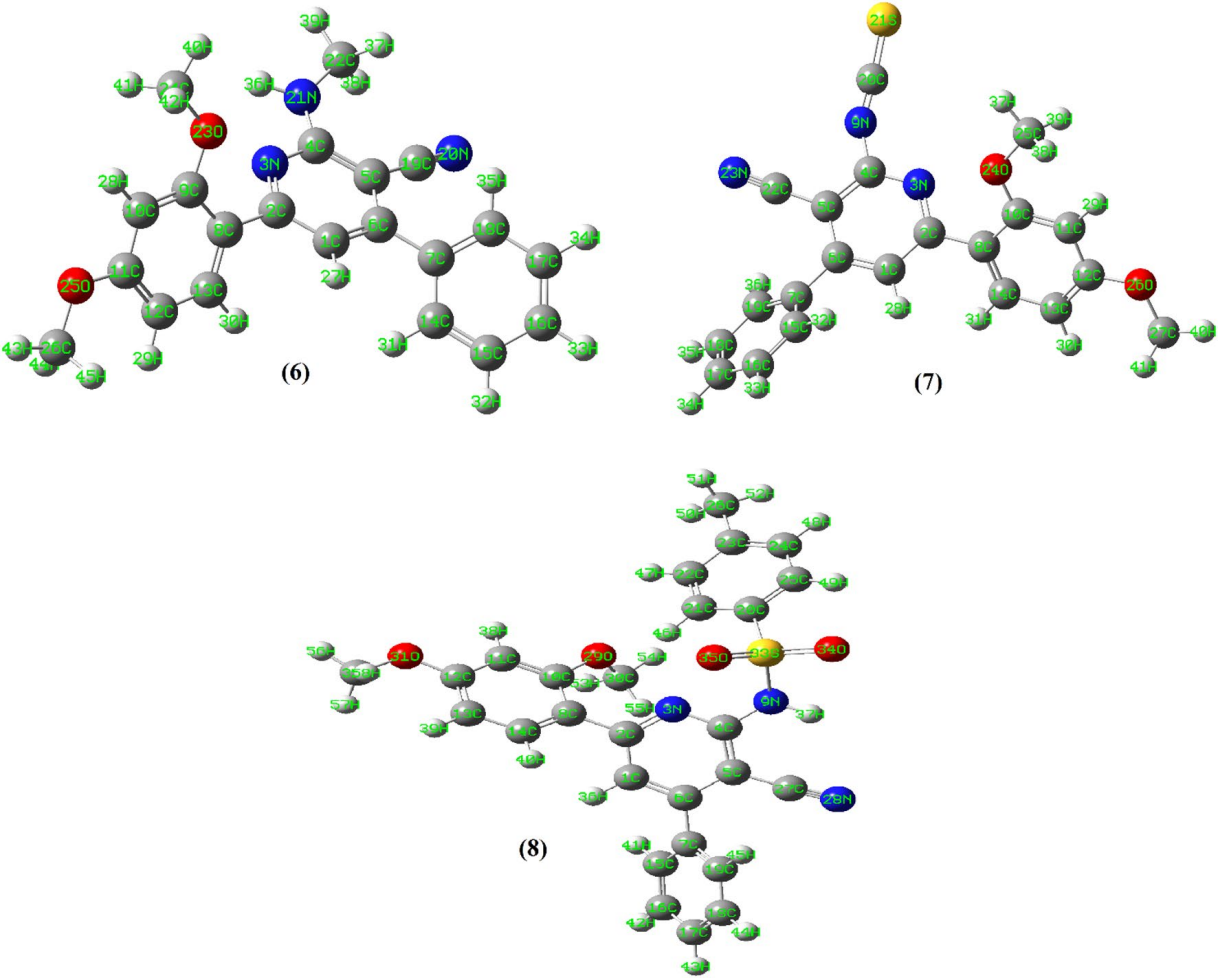
Geometrical optimize structures of compounds **6**–**8**.

Energetically optimized configurations of compounds **6**–**8** (minimum energies are 48.55, 46.34 and 228.17 kcal/mol, respectively) showed non-planar arrangements of the compounds, Fig. 11. Compound **8** showed high steric minimum energy may be due to the presence of the Ar–SO_2_ moiety. The planes containing the pyridine ring and the other phenyl parts in the compounds formed dihedral angles in the ranges of 40–50°. In the case of compound **6**, the NH group attached to the position 2 of pyridine is lying approximately in the same plane with the pyridine ring itself (dihedral angle of N3–C4–N21–H36 = 178.8°). In addition, the proton of the NH made intramolecular H-bonding with the pyridine nitrogen (N3–H36) with a distance equal to 2.29 Å, and consistent with the spectroscopic data. The optimized structure compound **7**, showed that the N=C=S group was linear and formed a bent angle with the pyridine plane (N3–C4–N9 = 116.99°), which deviated from the angle 120° characteristic for *sp*^*2*^ hybridization. No hydrogen bonding was detected in the structure of **7**. However, the other values of bond lengths and angles were found to be normal^38–40^. The energy optimized structure of compound **8** showed that sulfur atom (S33) of HN–SO_2_ group (Fig. 11) existed in a distorted tetrahedral arrangement. The bond angles involved in this tetrahedral such as O34–S33–O35 and N9–S33–O35 were 119.6° and 110.3°, respectively. In contradictory to compound 6, this structure arrangement forced the proton of the NH group to be away from the pyridine nitrogen. However, this proton acquired hydrogen bonding with the nitrogen of the cyano group (H37–N28 = 2.80 Å).

**Figure 12:**
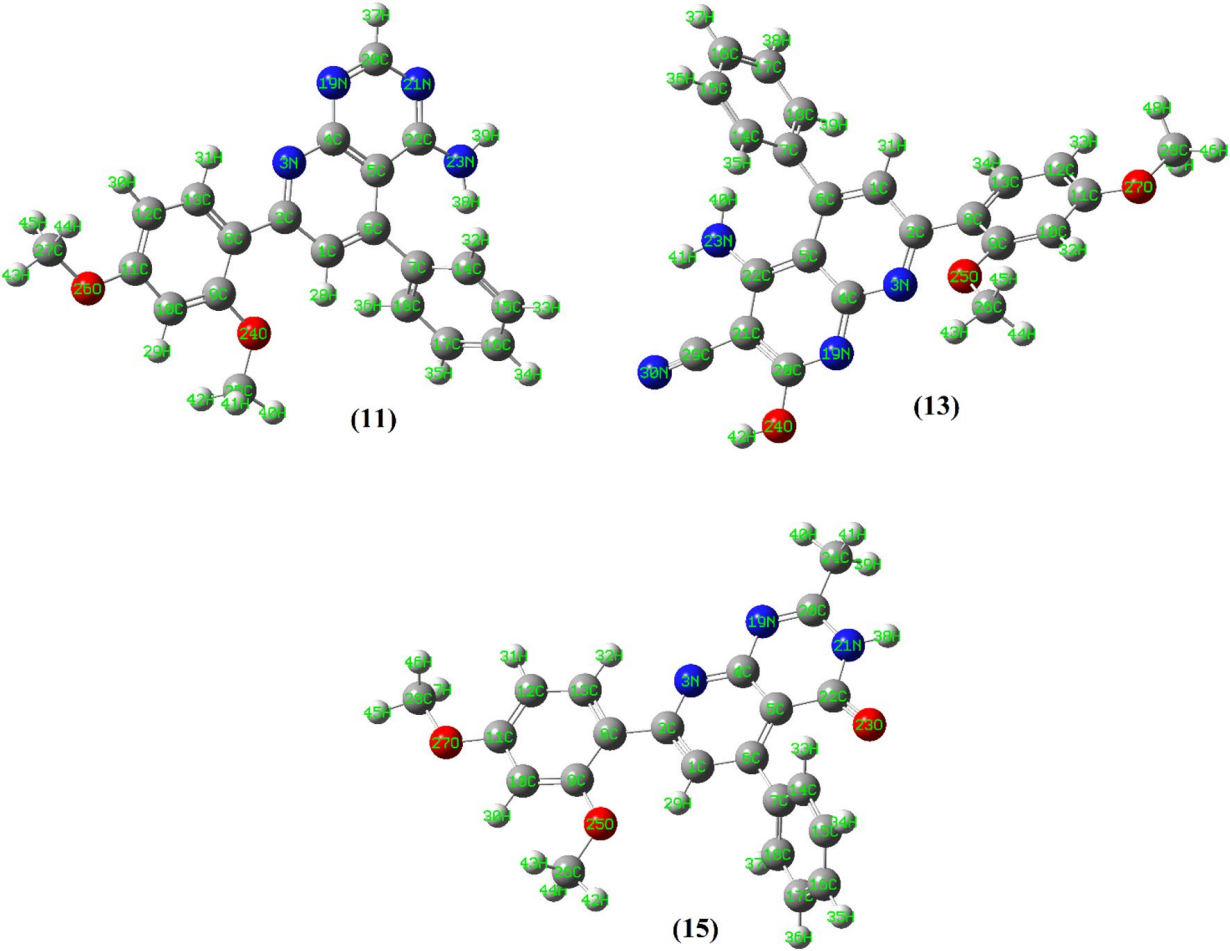
Geometrically optimize structures of compounds **11**, **13** and **15**.

The geometrically optimize configurations of the synthesized naphthyridine derivatives **11**, **13** and **15** are shown in Fig. 12. The minimization energies of the three compounds were 57.39, 59.60 and 50.33 kcal/mol, respectively. The optimized structure of **11** showed that it has unsymmetrical and non-planar geometry. The pyrido-pyrimidine, dimethoxy phenyl and amino groups were planar and lying in one plane, which made a dihedral angle (C1–C6–C7–C14) = 68.3° with the other phenyl ring. On the other hand, one of the amino protons made H-bonding with adjacent nitrogen of pyrimidine part (N21–H39 = 2.39 Å). For compound **13**, energetically optimized geometry showed that it was non-planar. The naphthyridine ring along with the amino, hydroxy and cyano groups exist in one plane and forming with both dimethoxy phenyl and the other phenyl groups dihedral angles with the values of 177.4° and 44.4°, respectively. On the other hand, the OH proton was directed towards the nitrogen atom of the cyano group (N30–H42) with van der Wall separation of 2.86 Å. One of the amino protons is also directed to the cyano group (N30–H41) with a distance equal to 2.88 Å. The optimized structure of compound **1** showed that the pyrido pyrimidinone moiety and the phenyl group of the dimethoxy phenyl part are planar. The other phenyl group exerted a dihedral angle with a value of 57.9° with that plane. The calculated structure displayed some sort of hydrogen bonding interaction between one of the nitrogen atoms of pyrimidinone ring and a hydrogen atom of adjacent –CH_3_ group (N19–H40 = 2.52 Å). Also, the carbonyl oxygen interacted with the adjacent NH proton (O23–H38) with a bond length of 2.43 Å. Further, the methoxy oxygen made H-bond interaction with the adjacent hydrogen of the pyrido group (O25–H29) with a value of 2.07 Å.

The chemical reactivity parameters such as the frontier *HOMO* and *LUMO* orbitals, energy differences gaps (*ΔE*), chemical potentials (*V*), ionization potentials (*I*), electronegativity (*Χ*) values, electron affinities (*A*), chemical softness (*S*) and hardness (*η*) values, as well as electrophilicity index (*ω*) amounts of the reported compounds are tabulated in Table 3^41,42^. Total energies, dipole moments (*DM*) and binding energies (*BE*) are also given. The DFT (B3LYP) calculations were performed to estimate the energies of the frontier molecular orbitals. The energy of the *HOMO* orbital describes the donation ability of the electrons and the energy of *LUMO* orbital refers to the withdraw capability of electrons. The difference in energies between the *HOMO* and *LUMO* (*ΔE*) points out to the molecular stability that demonstrates important parameters for the evaluation of the properties of electrical transport. The small differences in energy declare the facility of charge transfer (CT) as well as polarization in the compounds^36,38^. Derivative **13** had the smallest Δ*E* value. Moreover, the electronegativity factors reflect the electrostatic potentials, which show the partial transfer of the electron from a lowest electronegative to another highest electronegative part. The diminishing in electronegativities of the derivatives had the order: **3** > **4** > **2** > **8** > **7** > **13** > **5** > **11** > **15** > **1** > **6**. On the other side, results of small amounts of chemical hardness for the molecules reflected the capability of charge transfer within these compounds. The order of increasing charge transfer inside the molecules was: **5** < **6** ≈ **8** < **2** ≈ **3** ≈ **11** ≈ **15** < **4** < **7** < **1** < **13**. The binding energy (*BE*) represents the smallest amount of energy that required to disassemble a molecule into its constituent atoms. The values of binding energies can be calculated from the empirical relation^43^:

$$ BE = \frac{{\left[ {aE_{C} + bE_{H} + cE_{N} + dE_{O} + eE_{S} + fE_{Cl} } \right] - \left[ {E_{compound} } \right]}}{a + b + c + d + e + f} $$

*a*, *b*, *c*, *d*, *e* and f are the numbers of carbon, hydrogen, nitrogen, oxygen, sulfur or chloride in molecules. The *E*_C_, *E*_H_, *E*_N_, *E*_O_, *E*_*S*_ and *E*_Cl_ values represent the total energies of ground state for the different atoms. The *E*_compound_ exemplifies the energies of minimized structures of the calculated compounds. According to Table 3, order of decreasing *BE* of compounds was: **11** > **6** > **4** ≈ **5** > **3** > **15** ≈ **1** > **13** > **2** > **7** > **8**.

**Table 3:** The global chemical reactivity descriptors for the investigated compounds.

Parameter	**1**	**2**	**3**	**4**	**5**	**6**	**7**	**8**	**11**	**13**	**15**
Total Energy (au)	− 1086.8	− 1698.9	− 1331.6	− 1391.9	− 1278.6	− 1126.0	− 1521.8	− 1905.4	− 1180.2	− 1331.6	− 1239.4
*DM* (Debye)	6.80	11.30	12.47	12.56	8.45	4.80	8.99	9.67	6.30	8.47	2.33
*HOMO* (eV)	− 5.56	− 6.22	− 6.28	− 6.23	− 5.88	− 5.58	− 6.07	− 6.24	− 5.72	− 5.77	− 5.70
*LUMO* (eV)	− 1.61	− 2.16	− 2.23	− 2.20	− 1.54	− 1.40	− 2.07	− 2.05	− 1.66	− 1.90	− 1.64
*ΔE* (eV)	3.95	4.06	4.06	4.03	4.34	4.18	4.00	4.19	4.06	3.87	4.06
*Χ* (eV)	3.58	4.19	4.25	4.21	3.71	3.49	4.07	4.14	3.69	3.83	3.67
*V* (eV)	− 3.58	− 4.19	− 4.25	− 4.21	− 3.71	− 3.49	− 4.07	− 4.14	− 3.69	− 3.83	− 3.67
*A* (eV)	1.61	2.16	2.23	2.20	1.54	1.40	2.07	2.05	1.66	1.90	1.64
*I* (eV)	5.56	6.22	6.28	6.23	5.88	5.58	6.07	6.24	5.72	5.77	5.70
*η* (eV)	1.98	2.03	2.03	2.01	2.17	2.09	2.00	2.09	2.03	1.94	2.03
*S* (eV)	0.99	1.02	1.01	1.01	1.08	1.05	1.00	1.05	1.02	0.97	1.02
*ω* (eV)	3.25	4.31	4.46	4.41	3.17	2.92	4.14	4.10	3.35	3.79	3.32
*BE* (kcal/mol)	262.15	260.53	262.22	261.64	262.63	262.68	258.42	257.57	263.31	261.93	262.17

### Biological activity studies

#### The antimicrobial activities

Results of antimicrobial activities of screened compounds **1**–**8**, **11**, **13** and **15** showed that they have diverse antibacterial activities with respect to the standard Cefoperazone that varied according to the screened microbial strain and the tested compound (Tables 4 and 5). The investigated compounds displayed, with different ranges, abilities to inhibit the metabolic development of the screened bacteria and pointed out that they possess wide-spectrum properties. The various structures and substituents of the tested compounds obviously functioned an important role and considered the key pillar for their biological activity (vide infra). The activity of the reported compounds might be attributed to the existence of NH_2_, NH, OH, C=O and C≡N functional moieties. It was suggested that the mode of action of compounds is the formation of H-bond interaction between these groups and active places of cell constitutes along with interference with normal cell^43–45^. The cell membranes consist of permeable lipid layers, which allow the passage of soluble lipid materials. Therefore, the lipophilicity characteristics play a vital parameter that affects inhibition of bacteria. Thus, increasing the lipophilicity might enhance inhibition ability of screened compound. On the other hand, the tested compounds exhibited completely different trend for the antifungal activity studies. Except for the two compounds **2** and **9**, all the other screened compounds were inactive against *A. flavus, A. niger* and* F. oxysporum*. Compound **2** showed a satisfactory antifungal activity against *A. niger* (inhibition zone = 10 mm), while compound **9** showed the highest active towards *F. oxysporm* (inhibition zone = 15 mm).

**Table 4:** Correlation between the antibacterial activities and the structures of compounds 1–8.

No	Structure		Antibactrial activity (inhibition zone, mm)				
	Compound	Fragment	Gram + ve bacteria		Gram −ve bacteria		
			*S. aureus*	*B. cereus*	*E. coli*	*S. typhmirum*	*Y. enterocolitica*
	Cefoperazone	–	12	12	15	11	12
**1**			–	–	–	10	–
**2**			–	–	–	9	10
**3**			–	–	12	9	12
**4**			–	–	–	12	9
**5**			15	–	9	15	–
**6**			18	–	10	–	13
**7**			–	9	8	10	–
**8**			–	9	–	12	–

**Table 5:** Correlation between the antibacterial activities and the structures of compounds 11, 13 and 15.

No	Structure		Antibactrial activity (inhibition zone, mm)				
	Compound	Fragment	Gram + ve bacteria		Gram −ve bacteria		
			*S. aureus*	*B. cereus*	*E. coli*	*S. typhmirum*	*Y. enterocolitica*
	Cefoperazone	–	12	12	15	11	12
**11**			–	14	12	13	–
**13**			–	13	10	13	–
**15**			–	9	–	10	10

#### DPPH radical scavenging assay

**Figure 13:**
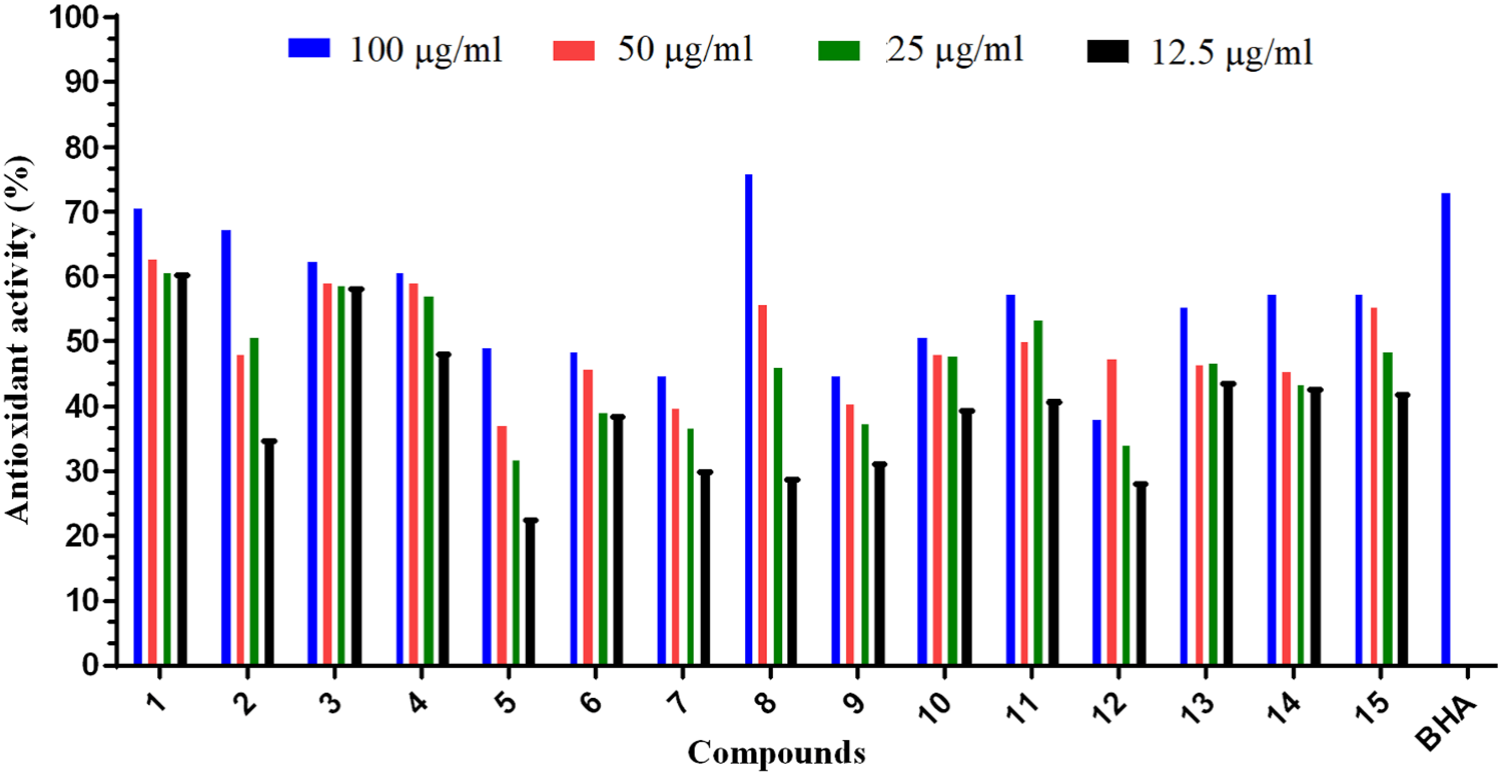
The DPPH radical scavenging activity of the reported compounds (**1**–**15**). Values are expressed as mean (n = 3) of the percent inhibition of the absorbance of DPPH radicals.

Normal physiology of living organisms is usually engaged with free radicals. Excessive amount of free radicals along with reactive oxygen are responsible for the induction of cellular oxidative damage. This damage in the cells would result in varieties of chronic diseases like for example arteriosclerosis, cancer, inflammatory disorders, and geriatric disorder^46^. The free radical agent 2,2-Diphenyl-1-picrylhydrazyl (DPPH) is widely used to estimate scavenging ability of various samples. Its significant decrease is dependent on the exposition to scavengers with proton radicals^47^. Thus, scavenging of the stable DPPH radical is valuable in determining antioxidant activity of a compound in a relatively short period. DPPH radical is little affected by side reactions like metal chelation and enzyme inhibition. This might be due to the extensive delocalization of its unpaired electron^48^. A stable diamagnetic radical is formed when the DPPH neutralize by an electron or a hydrogen^26^. DPPH gives deep violet color and displays a strong absorption band in the visible region (λ_max_ = 517 nm) corresponding to its unpaired electron. In presence of scavenging reagent, the electron paired up, absorption diminishes, and color turns to yellow. The decolorization is a stoichiometric step and it is relative to the number of electrons picked up. This reduction capability of DPPH radicals is induced by antioxidant agents and is estimated by lowering the absorbance of λ_max_ at 517 nm^49^. Figure 13 displays the DPPH radical scavenging activities of the investigated compounds (**1**–**15**). The commercial phenolic antioxidant, butylated hydroxyanisole (BHA) was used as a standard. As can be seen from Fig. 13, scavenging of DPPH radicals are dependent on concentration of the synthesized compounds. At 100 µg/ml, the two compounds (**5** and **12**) exhibited the lowest antioxidant activity, while compound **8** gave the highest scavenging activity (IC_50_ 43.39 µg/ml) against the DPPH radical. The obtained scavenging activity was raised by increasing the compounds concentrations ranged from 12.5 to 100 µg/ml. This increasing in scavenging activity is harmonized with the decrease in the DPPH radical concentration. It also indicated the reducing ability of DPPH radical by potential antioxidant properties of the reported compounds.

#### Structure–activity relationship

Results of this study declared the existence of relations between the antimicrobial actions of the reported derivatives and the different types of the substituents bonded to the enaminonitrile pyridine derivatives. It is worth mentioning that the substitutions varieties and the structure diversity of the tested compounds played an outstanding role in their biological activities. From the reactivity descriptors (Table 3), the data of antibacterial activity (Tables 4 and 5) and the antioxidant studies (Fig. 13), correlation between the structures and activities of the reported molecules can be achieved. The structure arrangement of a molecule is a reflection of its activity, i.e., analogous compounds could have similar activities. This fact is the central backbone of structure–activity relationship (SAR) proposition. The SAR concept thus supposes that structural characteristics of a molecule, such as its geometric and electronic properties have features responsible for its chemical, physical, and biological features. So, the SAR can be employed to speculate the biological activity of a compound from its structure arrangements and its substituents. Now, this approach is commonly applied in drug design and drug discovery to guide the development of the desired new compounds. As can be seen from Table 4, compound **1** showed antibacterial activity towards the Gram-negative bacteria *S. typhmirum* with inhibition activity zone = 10 mm. On substitution of NH_2_ of **1** by the HN–COCH_2_Cl moiety to yield compound **2**, the antibacterial activity against the Gram-negative bacteria enhanced and showed in hibition activity toward to the bacteria strains (*S. typhmirum* and *Y. enterocolitica*). Further, replacement of the fragment HN–COCH_2_C≡N to give compound **3**, i.e., C≡N instead of Cl in compound **2**, more enhancement in its antibacterial activity against the Gram-negative bacteria was indicated. Obviously, these fragments increased the lipophilic properties with the order **1** < **2** < **3**. Interestingly, this was the order of the calculated electrophilicity index, chemical potential, and chemical softness of the three derivatives (Table 3). In the case of compound **4**, the substituted branched group, N(COCH_3_)_2_, instead of NH_2_ caused inhibition disability and reduced its antibacterial activity. On the other hand, replacement of the NH_2_ group of compound **1** by the fragments N = OEt, NHCH_3_ and N=C=S to give the compounds **5**–**7**, enhanced the antibacterial ability of the compound against the Gram-positive bacteria with high inhibition zones (Table 4). These data are also correlated with the reactivity descriptors of these derivatives (Table 3) as well as antioxidant activities (Fig. 13). The interaction between inhibitors, such as synthetic compounds and bacteria could be due to either diffusion or cell permeability. Thus, the cell wall of a bacteria plays crucial role against the screened inhibitor. The cell wall of the Gram-positive bacteria mainly consisted of multiple layers of peptidoglycan that forms a thick (cell wall = 20–30 nm) and rigid membrane structure, while the cell wall of the Gram-negative bacteria has an outer membrane and fewer layers of peptidoglycan (cell wall is 8–12 nm thick)^50^.

Reactions of compound **1** with formamide, N≡C–CH_2_–COOEt and Ac_2_O + H_2_SO_4_ gave different category of products (naphthyridine derivatives **11**, **13** and **15** (Table 5). The reported naphthyridine derivatives were expected to have important biological applications^32–34^. These interesting cyclic products showed fair antibacterial inhibition with comparable activities towards the Gram-negative bacteria and one of the Gram-positive bacteria (*B. cereus*). They also have almost analogous antioxidant activities (Fig. 13). Interestingly, the reported compounds (**11**, **13** and **15**) have also comparable calculated reactivity descriptors (electrophilicity index, chemical potential and chemical softness, Table 3.

### Molecular docking studies

**Figure 14:**
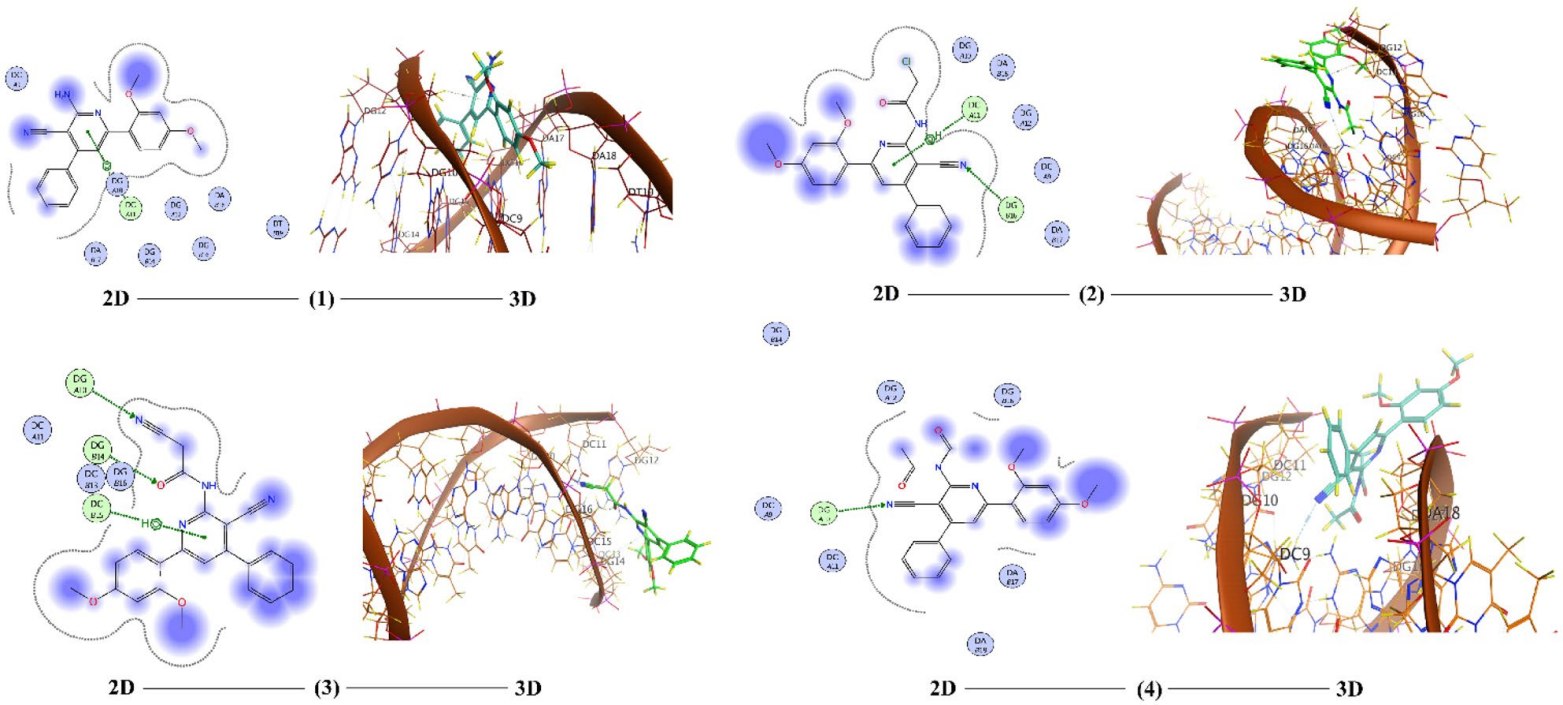
The 2D and the 3D representations of the interaction of investigated compounds **1**–**4** with DNA.

**Figure 15:**
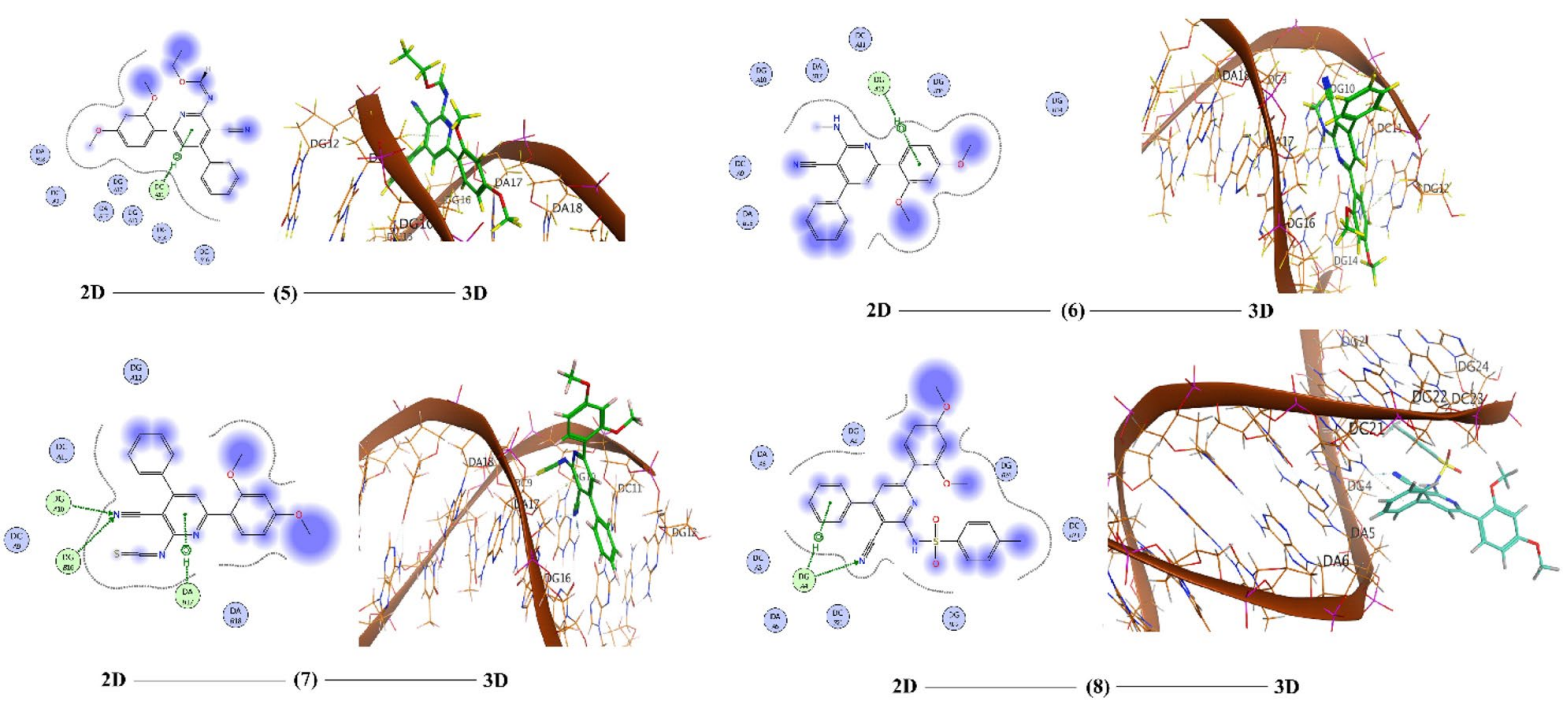
The 2D and the 3D representations of the interaction of investigated compounds **5–8** with DNA.

**Figure 16:**
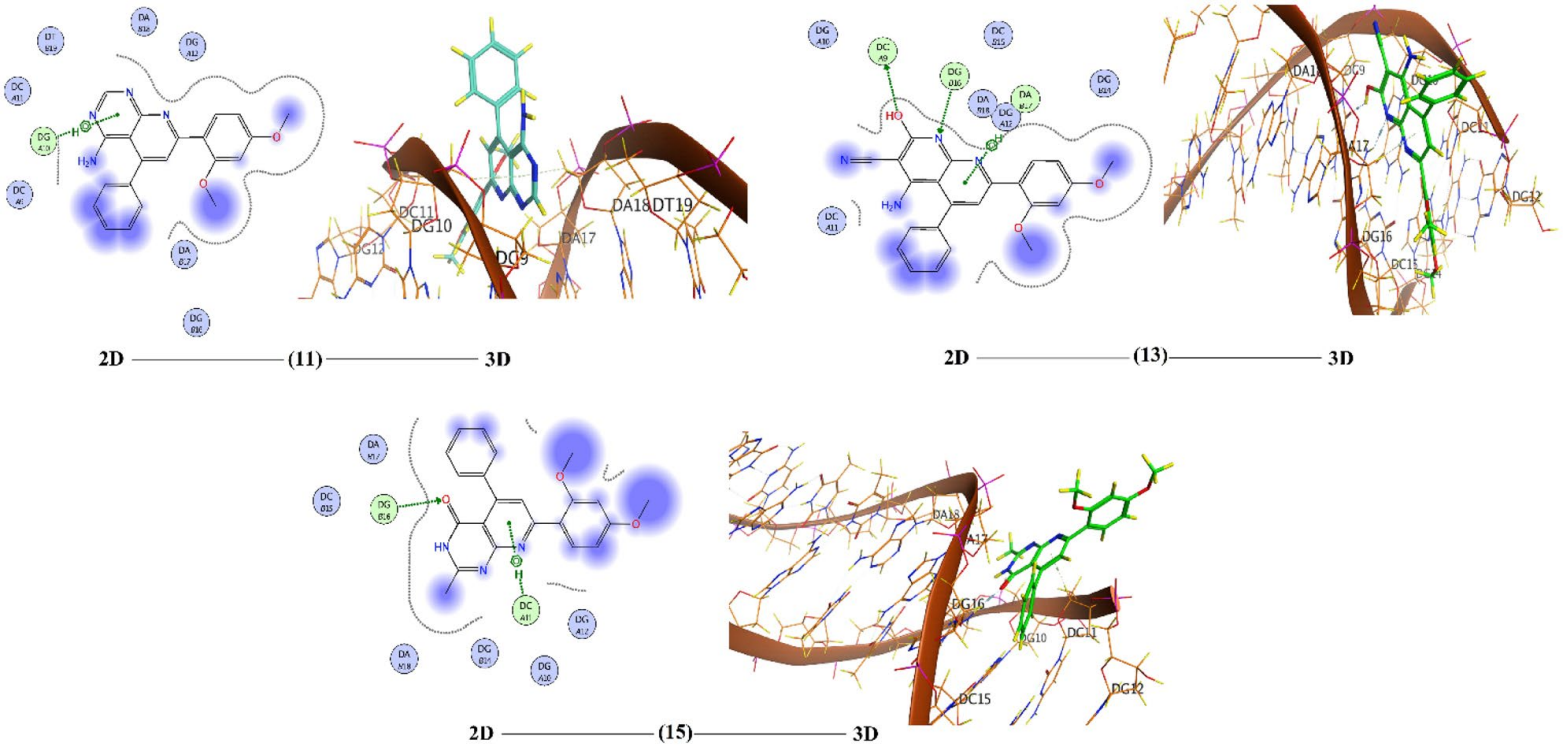
The 2D and the 3D representations of the interaction of investigated compounds **11**, **13** and **15** with DNA.

Computation of molecular docking is a superior rout to estimate the interaction between small, prepared molecules and macromolecular biological targets^51^. Analyses of the docking data are commonly utilized in detecting the conformation variations that associated with the binding sites of macromolecular receptors, like nucleic acid, to host the docked hydrophobic inhibitor. Molecular docking analysis of the compounds **1**–**8**, **11**,** 13** and **15** were executed by using MOE program. The investigation intended to declare the types of interactions between the tested compounds and DNA as well as to distinguish between the possible binding poses and energy values. The conformations possibilities of the docked compound and a B-nucleic acid (PDB ID:1BNA) were valued from the binding scores, hydrophobic interactions, and different types of hydrogen bond formation. Molecular docking studies estimate the way to how docked molecules are fundamentally adjusted into the minor groove of the DNA and declare the type of interactions (binding poses) with the DNA bases, which could be either hydrophobic, ionic or hydrogen bonding (Figs. 14, 15 and 16). These binding cases are evaluated using the mathematical scoring function (S)^23^. These score functions values are usually negative. The large negative values of S imply that the docked compound has high binding affinity to bind with the macromolecular receptor. Notably, molecular docking analysis of the investigated derivatives displayed high negative values for the functions binding scores and confirmed the good efficiencies of these bioactive molecules towards the investigated target. Table 6 tabulates the computed docking findings (score and rmsd values, types and energies of interactions). As can be observed from Figs. 14, 15 and 16 and Table 6, the nucleotide regions DG, DC, and DA of the DNA receptor were the most optimum docking zones. Binding modes and poses were due to hydrophobic interactions between nucleotides residues like DA, DC, and DG and aromatic parts of the compounds. Further, hydrogen-donor, hydrogen-acceptor and/or π-H occurred between compounds and DNA parts were another modes of interactions. The binding interaction was found to have the order: **3** < **8** < **15** < **7** < **6** < **4** < **2** < **11** < **13** < **5** < **1**.

**Table 6:** The molecular docking data of the reported compounds.

Compound	S (kcal/mol)	rmsd value ^a^	Ligand-Receptor	Interaction type	Interaction distance, Å	E (kcal/mol)
**1**	− 8.54	1.50	6-ring-C4′ (DC 11A)	π-H	3.79	− 0.6
**2**	− 6.79	1.86	N31-N2 (DG 16B)	H-acceptor	3.49	− 1.5
			6-ring-C4′ (DC 11A)	π-H	4.03	− 0.6
**3**	− 4.66	1.32	O43-N2 (DG 14 B)	H-acceptor	3.15	− 2.0
			N48-N2 (DG 10 A)	H-acceptor	3.19	− 1.0
			6-ring-C4′ (DC 15 B)	π-H	4.44	− 1.2
**4**	− 6.56	1.41	N30-N2 (DG 10 A)	H-acceptor	3.34	− 2.9
**5**	− 8.19	2.36	6-ring-C4′ (DC 11 A)	π-H	3.85	− 0.9
**6**	− 6.50	2.01	6-ring-C5′ (DG 12 A)	π-H	4.44	− 0.6
**7**	− 6.46	2.37	N32-N2 (DG 10 A)	H-acceptor	3.13	− 3.7
			N32-N2 (DG 16 B)	H-acceptor	3.18	− 1.5
			6-ring-C4′ (DA 17 B)	π-H	3.82	− 0.8
**8**	− 5.23	1.69	N45-N2 (DG 4 A)	H-acceptor	3.19	− 0.8
			6-ring-N2 (DG 4 A)	π-H	4.13	− 0.7
**11**	− 7.24	1.46	6-ring-C5′ (DG 10 A)	π-H	3.55	− 1.1
**13**	− 8.01	1.55	O35-O2 (DC 9 A)	H-donor	3.01	− 2.1
			N28-N2 (DG 16 B)	H-acceptor	3.11	− 3.2
			6-ring-C4′ (DA 17 B)	π-H	3.69	− 0.7
**15**	− 6.25	1.40	O33-N2 (DG 16 B)	H-acceptor	2.98	− 4.1
			6-ring-C4′ (DC 11 A)	π-H	3.70	− 1.0

^a^The root mean square deviation; a measure of the average distance between the docked atoms.

## Conclusion

Green syntheses of fourteen molecularly designed pyridine derivatives were performed from the reactions of the enaminonitrile 2-amino-6-(2,4-dimethoxyphenyl)-4-phenylnicotinonitrile with some selected reagents. The density functional theory calculations for some selected compounds and their computed quantum global reactivity descriptors showed their interesting structural arrangements and their possible biological activities. The antimicrobial inhibition of the molecules that screened against four bacterial strains as well as their antioxidant activities indicated their potency as potential antibiotic reagents. The structure–activity relationships of these compounds were also correlated with the data of antibacterial and antioxidant activities. Furthermore, the molecular docking studies for some derivatives with a B-DNA nucleic acid (PDB ID:1BNA) illustrated high negative binding scores due to the presence of different hydrogen bonding interactions (H-acceptor, H-donor and π-H). Such interactions reflected their high binding affinities to the tested macromolecular receptor.

### Supplementary Information

Supplementary Information.

## Data Availability

Any datasets used that support the findings of this study are available from the corresponding author upon reasonable request. A supplementary file, containing the figures of ^1^H- and ^13^C-NMR of compound **1** and a table of the % yields of all products, is provided.
