# Supplementary material for: Synthesis, spectroscopic, DFT calculations, biological activity, SAR, and molecular docking studies of novel bioactive pyridine derivatives
Source: Sci Rep. 2023 Sep 20;13:15598. doi: 10.1038/s41598-023-42714-w (PMC10511440; doi:10.1038/s41598-023-42714-w)
Supplement: Supplementary file 1 — Supplementary Information. [file 41598_2023_42714_MOESM1_ESM.docx]

**Supplementary File**

**Synthesis, spectroscopic, DFT calculations, biological activity, SAR, and molecular docking studies of novel bioactive pyridine derivatives**

Kurls E. Anwer ^1^, Zeinab K. Hamza ^2^ and Ramadan M. Ramadan ^1,^*

*^1^ Department of Chemistry, Faculty of Science, Ain Shams University, Cairo, Egypt*

*^2^ Food Toxicology and Contaminants Department, National Research Centre, Egypt*

* Corresponding author: [r_m_ramadan@yahoo.com](mailto:r_m_ramadan@yahoo.com)


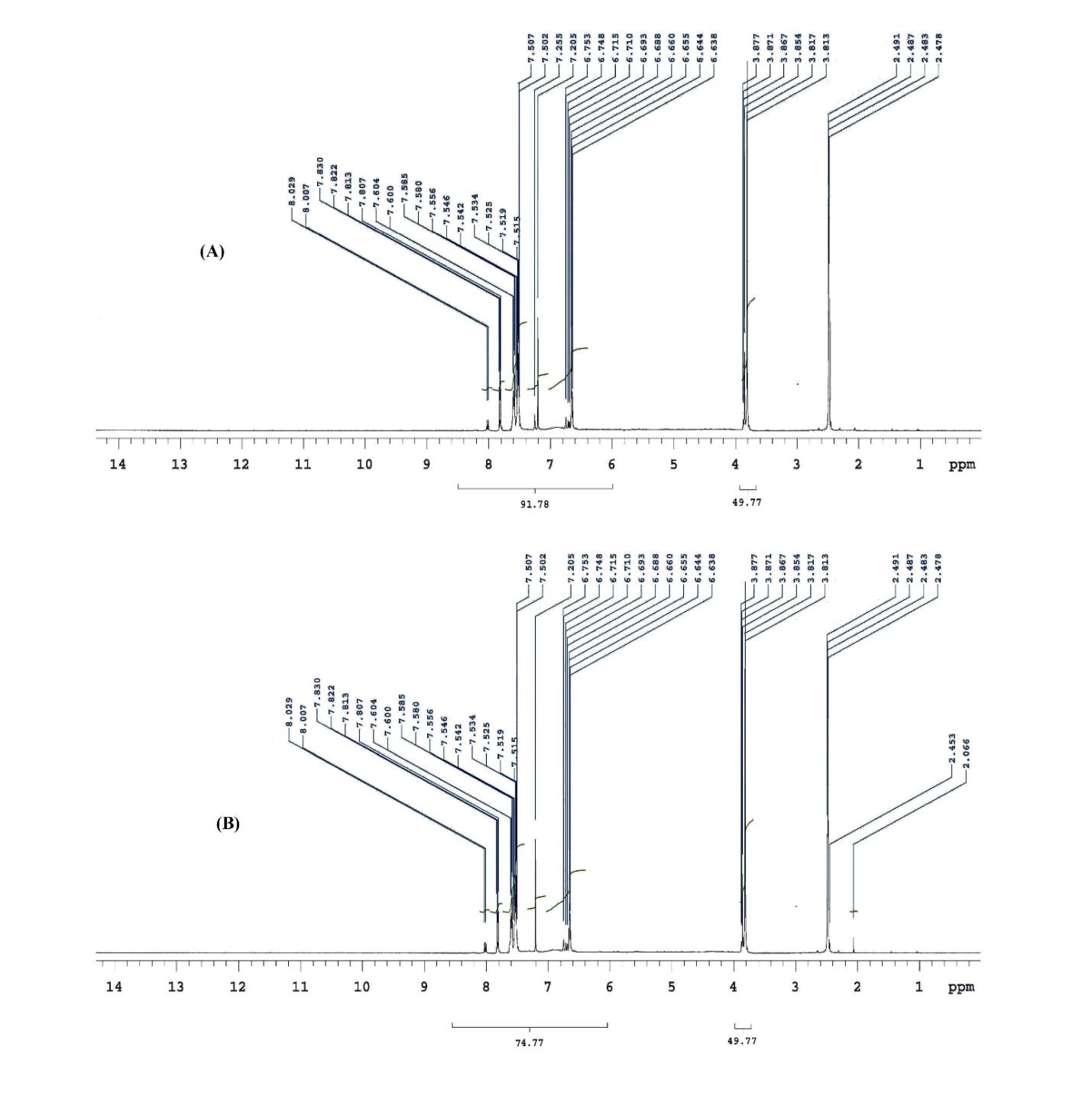


**Figure 1S. (A)** The ^1^H-NMR spectrum of **1**; **(B)** The ^1^H-NMR spectrum of **1** + D_2_O.


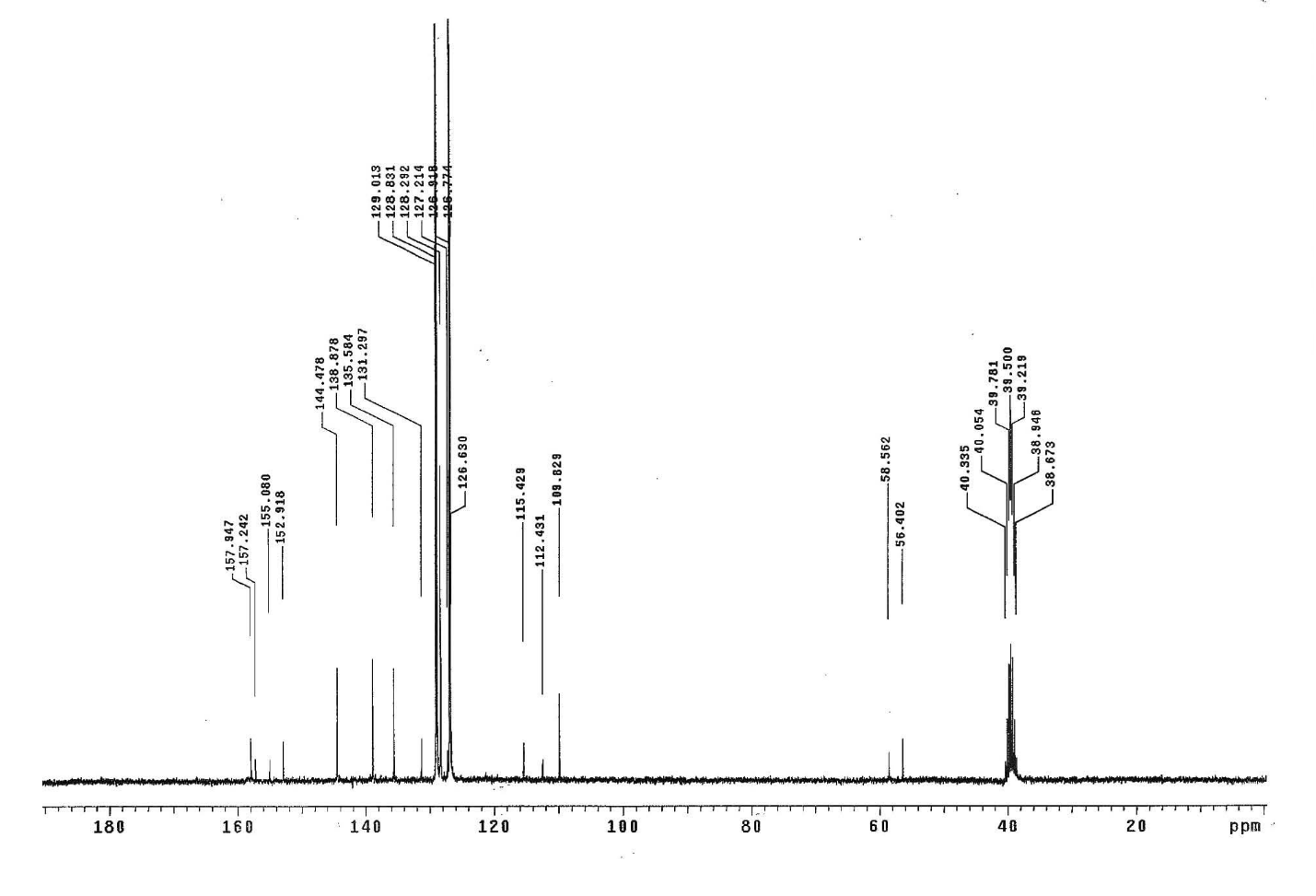


**Figure 2S.** The ^13^C-NMR spectrum of **1**.

**Table (1S).**  The comparison between the prepared target heterocyclic derivatives (1-27) under microwave and conventional technique in terms physical data.

| **Compound no.** | **Time (min)** | | **Yield (%)** | | **YE** | | **RME** | | **OE** | | **AE** |
| --- | --- | --- | --- | --- | --- | --- | --- | --- | --- | --- | --- |
|  | M.W. | Con. | M.W. | Con. | M.W. | Con. | M.W. | Con. | M.W. | Con. |  |
| **1** | 1 | 300 | 93 | 70 | 93 | 0.233 | 73.03 | 54.97 | 93 | 70 | 78.52 |
| **2** | 2 | 1080 | 90 | 72 | 45 | 0.067 | 70.99 | 56.79 | 77.27 | 61.82 | 91.87 |
| **3** | 2 | 720 | 91 | 77 | 45.5 | 0.107 | 76.09 | 64.38 | 79.53 | 67.29 | 95.67 |
| **4** | 2 | 1440 | 93 | 71 | 46.5 | 0.049 | 89.14 | 70.92 | 93 | 71 | 95.84 |
| **5** | 2 | 1200 | 90 | 74 | 90 | 0.062 | 72.71 | 59.79 | 90 | 74 | 80.79 |
| **6** | 3.5 | 1140 | 94 | 76 | 26.85 | 0.067 | 64.35 | 52.02 | 79.27 | 65.03 | 81.18 |
| **7** | ----- | 1680 | ----- | 77 | ----- | 0.046 | ---- | 70.57 | ---- | 77 | 91.65 |
| **8** | 2 | 600 | 90 | 70 | 45 | 0.117 | 73.36 | 57.06 | 78.81 | 61.30 | 93.09 |
| **9** | 2.5 | 960 | 88 | 74 | 35.2 | 0.077 | 73.96 | 62.20 | 77.20 | 64.93 | 95.80 |
| **10** | 1.5 | 1060 | 92 | 72 | 61.33 | 0.068 | 78.69 | 61.58 | 81.76 | 63.99 | 96.24 |
| **11** | 2 | 1200 | 87 | 73 | 43.5 | 0.061 | 82.84 | 69.51 | 87 | 73 | 95.21 |
| **12** | 2 | 1440 | 89 | 77 | 44.5 | 0.053 | 72.92 | 63.09 | 77.22 | 66.81 | 94.43 |
| **13** | 2 | 1060 | 87 | 73 | 43.5 | 0.069 | 65.46 | 54.93 | 74.72 | 62.70 | 87.61 |
| **14** | 2 | 600 | 90 | 71 | 45 | 0.119 | 75.86 | 59.84 | 75.86 | 59.84 | 100 |
| **15** | 2 | 900 | 90 | 72 | 45 | 0.800 | 77.53 | 62.02 | 90 | 72 | 86.14 |
